# Super-resolution imaging reveals altered actin architecture at CAR-T cell immune synapses

**DOI:** 10.1083/jcb.202506119

**Published:** 2026-08-05

**Authors:** Olivia P.L. Dalby, Efstratios Kirtsios, Hale-Seda Radoykova, Cecilia Zaza, Megan D. Joseph, Persis J. Amrolia, Alice Giustacchini, Sabrina Simoncelli

**Affiliations:** 1 London Centre for Nanotechnology, University College London, London, UK; 2 Department of Chemistry, University College London, London, UK; 3 Molecular and Cellular Immunology Section, Great Ormond Street Institute of Child Health, UCL, London, UK; 4 Human Technopole, Milan, Italy

## Abstract

The actin cytoskeleton plays a key integrative role in immunological synapse (IS) formation during T cell activation, but how these dynamics are altered in chimeric antigen receptor (CAR)-T cells remains unclear. Here, we used stimulated emission depletion (STED) microscopy to perform the first super-resolution analysis of actin remodeling at the IS in single- and dual (CD19/CD22) CAR-T cells, activated on supported lipid bilayers across different time points. Quantitative imaging reveals that CAR-T cells form structurally distinct synapses to untransduced cells, characterized by reduced actin-depleted regions, fewer actin foci, and persistent microvilli-like protrusions. These features indicate incomplete cytoskeletal contraction and impaired actin network reorganization, leading to partial synapse maturation. Our findings highlight fundamental differences in actin dynamics between CAR- and TCR-mediated signaling and suggest that defective actin remodeling may contribute to unstable synapse formation, altered signaling integration, and dysregulated responses or off-target effects. These insights could inform future CAR-T engineering strategies to enhance safety and efficacy.

## Teaser

Super-resolution imaging reveals that CAR-T cells assemble immunological synapses with distinct actin architectures, including reduced central clearance and enhanced protrusions, highlighting fundamental cytoskeletal differences from TCR-driven synapses that may shape CAR-T signaling dynamics, synapse stability, and effector function.

## Introduction

Chimeric antigen receptor (CAR) T cell therapy represents a major breakthrough in cancer immunotherapy, enabling genetically engineered T cells to recognize and eliminate tumor cells. CARs are synthetic immunoreceptors composed of an extracellular antibody-derived single-chain variable fragment (scFv), a hinge and a transmembrane domain, and an intracellular CD3ζ signaling domain. This configuration mimics key aspects of T cell receptor (TCR) signaling to drive cytotoxic responses against cancer cells. Since the initial demonstration of CAR-mediated T cell activation in the early 1990s (Kuwana et al., 1987; Gross et al., 1989; Irving and Weiss, 1991), successive generations of CARs have been developed to improve efficacy, broaden target specificity, and reduce off-target toxicity (June et al., 2018; Chen et al., 2023; Liu et al., 2020).

While these efforts have been accompanied by numerous clinical trials, the formation and structural characteristics of the immunological synapse (IS) in CAR-T cells remains unclear, particularly when compared with the canonical TCR-mediated IS (Li et al., 2020). In TCR-mediated activation, synapse formation is tightly coordinated by the actin cytoskeleton, which drives cell spreading, receptor clustering, and signaling integration (Mastrogiovanni et al., 2020). This results in a highly organized structure composed of concentric supramolecular activation clusters (SMACs) (Roy and Burkhardt, 2018): a distal actin-rich ring (dSMAC), a peripheral contractile actin arc zone (pSMAC), and a central actin–depleted region (cSMAC), where signaling complexes accumulate and are eventually downregulated (Varma et al., 2006).

Key regulators of this cytoskeletal remodeling include the Arp2/3 complex, formins, and actin-binding proteins, such as WASp and WAVE2, which control the geometry and dynamics of filament networks (Fritzsche et al., 2017; Babich et al., 2012; Ashdown et al., 2017; Dustin et al., 2010). Additionally, TCR signaling microclusters translocate centripetally via retrograde actin flow, while costimulatory receptors and integrins anchor the cytoskeleton to stabilize the synapse. These spatially resolved actin structures are essential for antigen sampling, mechanical force generation, and directional secretion of cytotoxic granules.

In contrast, emerging evidence suggests that CAR-T cells form synapses that diverge structurally and functionally from the canonical TCR paradigm. Confocal studies by Davenport et al. (2018) and Xiong et al. (2018) revealed that CAR-T cells, including anti-HER2 and κ-CARs, exhibit more irregular synapse morphologies compared with untransduced T cells, including reduced central actin clearance and disorganized Lck signaling clusters. Consistent with these observations, Dong et al. (2020) further reported that CAR-T cells display limited central F-actin clearance. Intriguingly, the same study demonstrated that CAR signaling can proceed independently of linker for activation of T cells, a scaffold protein essential for TCR-mediated actin reorganization, suggesting that CARs engage a rewired signaling network to coordinate cytoskeletal dynamics and activation. Together, these studies suggest that although CAR-T cells form functional synapses, their cytoskeletal architecture and underlying signaling mechanisms may be fundamentally distinct from those of TCR-activated T cells, more closely resembling innate cytotoxic cells such as natural killer cells. Importantly, these findings are based primarily on diffraction-limited imaging and largely assess mature synapses at late activation time points (e.g., ≥10–20 min after activation), offering limited insight into the early and intermediate dynamics of synapse formation. Moreover, how CAR engagement affects the spatiotemporal organization of actin—particularly during the transition from initial contact to synapse maturation—remains poorly defined.

To address these gaps, here we apply super-resolution stimulated emission depletion (STED) microscopy, a cutting-edge fluorescence microscopy technique capable of achieving ∼60 nm resolution, to visualize actin remodeling during the activation of primary T cells, as well as single- and dual (CD19/CD22) CAR-T cells. By capturing high-resolution snapshots at key time points (1, 3, and 8 min), we reveal that CAR-T cells undergo rapid cytoskeletal changes characterized by (1) more abrupt formation of outer F-actin rings, (2) smaller actin-depleted central zones, (3) fewer actin foci, (4) sustained microvilli-like protrusions, and (5) enlarged actin meshwork. Consistent with these data, high-resolution live-cell imaging of F-actin dynamics also confirmed that, unlike untransduced T cells, CAR-T cells maintain elevated peripheral projections over time, suggesting a failure to complete the canonical spreading–contraction cycle and resulting in a disorganized or partially matured synaptic architecture. Altogether, these features suggest that CAR-T cells form structurally distinct synapses, with implications on signaling fidelity, granule delivery, and cytotoxic function.

## Results and discussion

### Dual CAR-T cells activate more efficiently than single CARs at low antigen densities

Our study focuses on a dual-transduction approach using CAT-CD19-BBZ and 9A8-CD22-BBZ CAR-T cells, which are currently under evaluation in a phase 1 clinical trial for relapsed/refractory pediatric B-lineage acute lymphoblastic leukemia (NCT02443831). This dual CAR-T cell strategy designed to reduce risk of antigen escape, especially the common CD19-negative relapse, by simultaneously targeting CD19 and CD22 (Kokalaki et al., 2023). Notably, the anti-CD19 CAR (CAT-CD19-BBZ) features lower-affinity binding domain (K_d_ = 14 nM) (Ghorashian et al., 2019), previously associated with reduced toxicity relative to the licensed higher-affinity constructs (Ghorashian et al., 2019). Whereas the anti-CD22 CAR (9A8-CD22-BBZ) has high affinity for its ligand (K_d_ = 1.9 nM) (Kokalaki et al., 2023). Despite the clinical promise of multitargeting CAR-T cell strategies, little is known about how co-expression of two distinct CARs with distinct affinities for their respective antigens in the same T cell impacts cytoskeletal remodeling during synapse formation.

**Figure 1.**
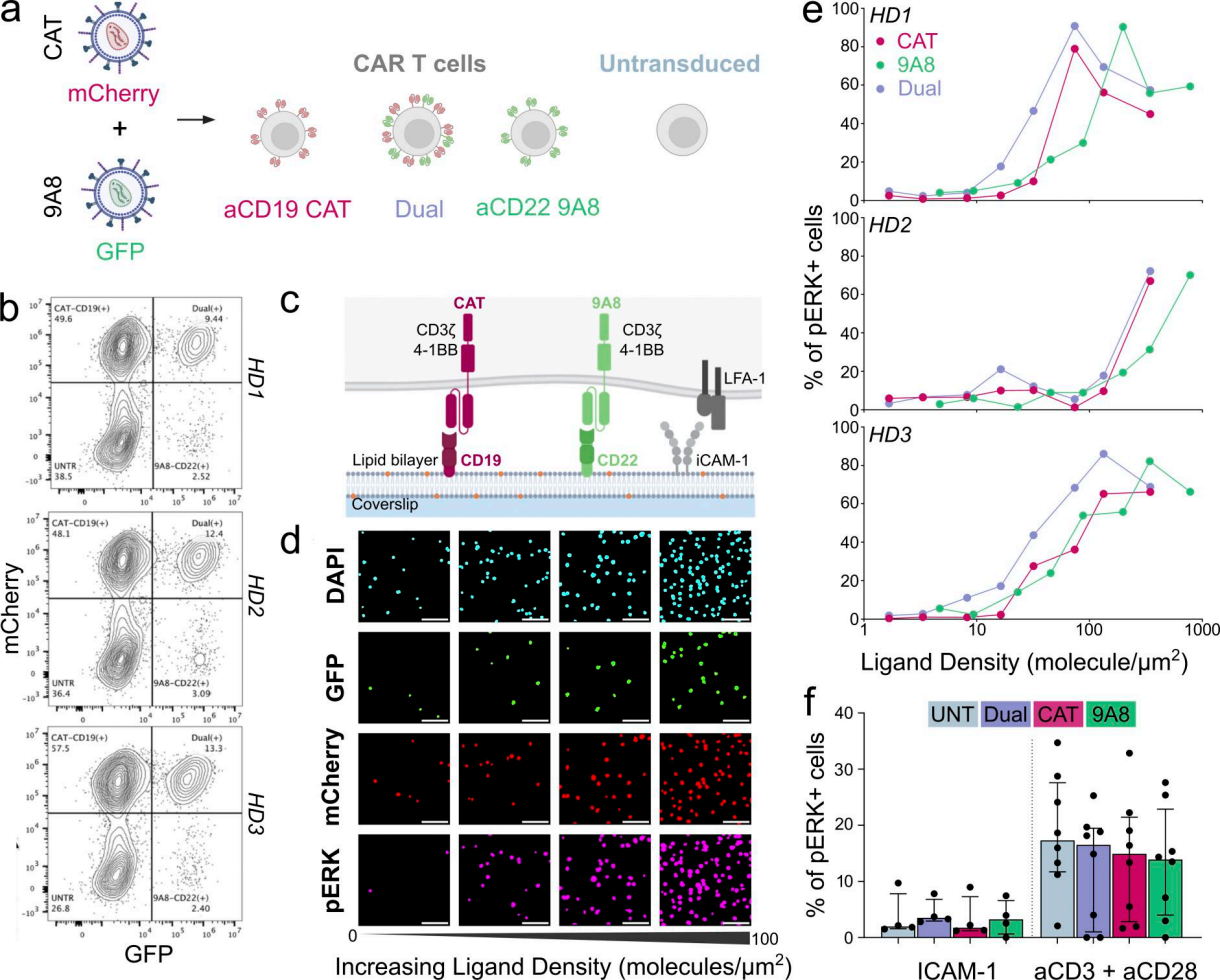
**Dual CAR-T cells activate more efficiently than single CAR-T cells at low antigen densities.** **(a)** Schematic of CAR-T cell production from healthy donor PBMC samples (*n* = 3 biological donors) with mCherry-CAT-CD19-BBZ-CAR and eGFP-9A8-CD22-BBZ-CAR lentiviral vectors producing four different cell types: untransduced T cells (blue), aCD19 CAT CAR-T cells (red), aCD22 9A8 CAR-T cells (green), and dual aCD19 CAT and aCD22 9A8 CAR-T cells (purple). **(b)** FACS results to measure the transduction efficiency of the three healthy donor PBMC samples (HD1, HD2, and HD3) with mCherry-CAT-CD19-BBZ-CAR and eGFP-9A8-CD22-BBZ-CAR lentiviral vectors. **(c)** Schematic of SLB system functionalized with biotinylated and His-tagged CD19 and CD22, and His-tagged ICAM-1, interacting with a dual 9A8 and CAT CAR-T cell. **(d)** Confocal images of CAR-T cells stimulated with the SLB at increasing ligand densities of CD19 and CD22 in molecules/µm^2^. Showing DAPI staining for all cells (blue), GFP staining for 9A8-positive CAR-T cells (green), mCherry staining for CAT-positive CAR-T cells (red), and pERK staining for activated cells (pink). Scale bar is 250 µm. **(e)** Analysis of confocal pERK data representing % of cells expressing pERK at each ligand density on the SLB for CAT (red), 9A8 (green), and dual (purple) CAR-T cells for HD1, HD2, and HD3 biological donors. CAT and dual CAR T cells were plotted against CD19 ligand density, while single 9A8 CAR T cells were plotted against CD22 density. Each datapoint is from one biological donor with cells taken from 10 fields of view (FOVs), with a separate graph for each biological donor (HD1, HD2, and HD3). **(f)** Analysis of confocal pERK data representing % of cells expressing pERK interacting with SLB functionalized with ICAM-1 or anti-CD3/anti-CD28 with ICAM-1 on the SLB for untransduced T cells (blue), and dual (purple), CAT (red), and 9A8 (green) CAR-T cells. Each black circle represents one experimental repeat from a different biological donor, with bars representing median with interquartile range shown as error bars. Statistical comparisons were performed using two-tailed nonparametric Mann–Whitney U tests (Wilcoxon rank-sum tests), with Benjamini–Hochberg (BH) correction for multiple comparisons. P values above 0.05 were considered not significant. FACS, fluorescence-activated cell sorting.

To explore these questions, we analyzed a mixed CAR-T product—composed of both single and dual CAR-expressing cells—reflecting the cellular heterogeneity typically observed in clinical co-transduction manufacturing settings. This product included three distinct CAR-T cell populations: cells expressing CAT-CD19-BBZ alone (mCherry^+^), 9A8-CD22-BBZ alone (eGFP^+^), and double-positive cells (mCherry^+^/eGFP^+^), generated via double lentiviral transduction of peripheral blood mononuclear cells (PBMCs) from healthy donors (*n* = 3 biological donors) (Fig. 1 a). Both CAR constructs contain a 4-1BB costimulatory domain and a CD3ζ activation domain. Untransduced T cells from the same donors were used as controls.

**Figure S1.**
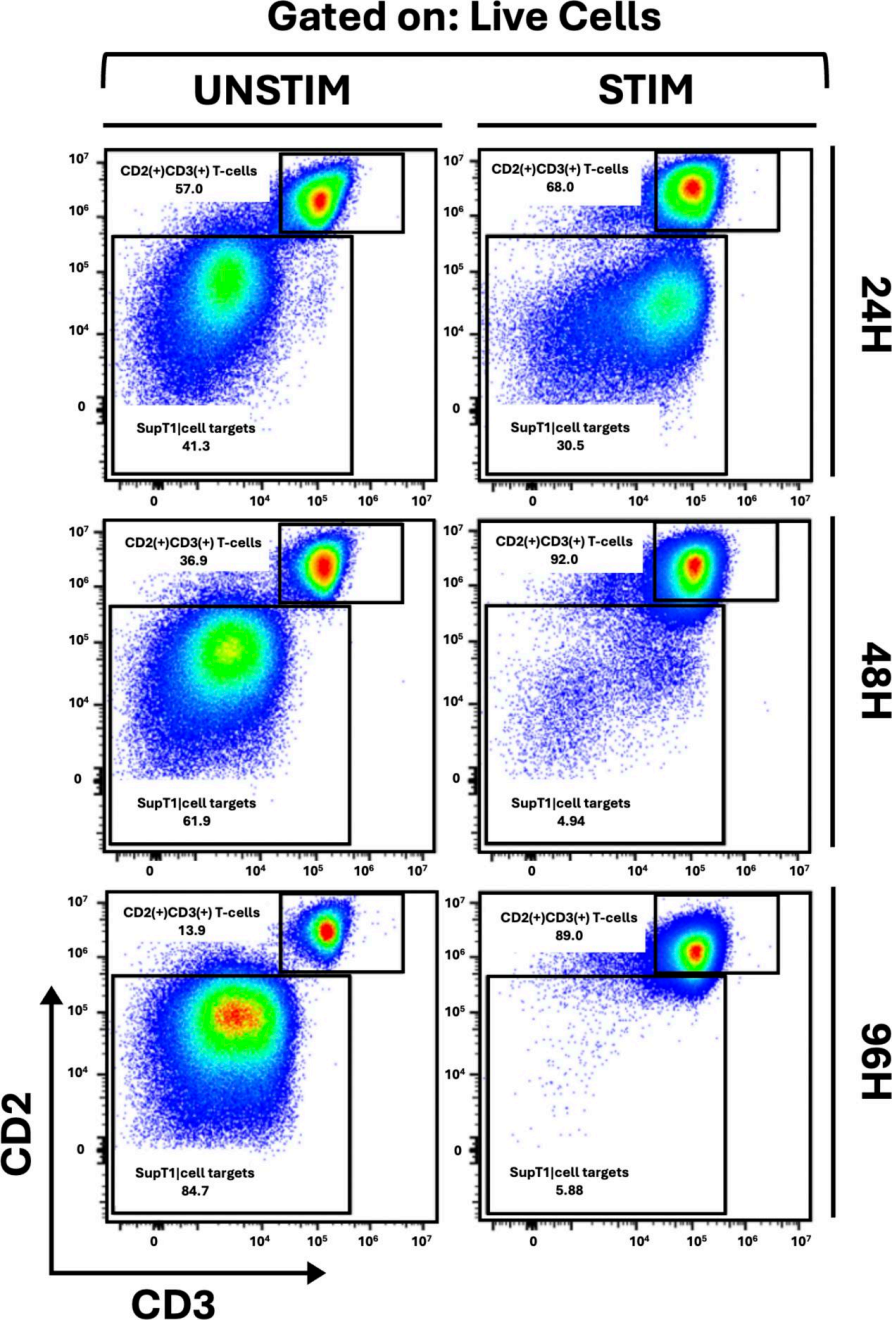
**Co-culture assay to confirm cytotoxic ability of dual CAR-T cell products.** UNSTIM-condition is dual CAR-T cell product in co-culture with nonirradiated SupT1-WT cell line as a non-stimulation control. STIM-condition is non-irradiated SupT1-CD19/CD22, SupT1 cells engineered to express the pan–B cell CD22 and CD19 markers, serving as antigen expressing target cells to stimulate dual–CAR-T cell products. Two pan–T cell markers, CD2 and CD3, were used to distinguish SupT1-targets from effector CAR T cells via FACS since the SupT1 lack CD2 expression (ATCC, CRL-1942) and CD3 surface expression. CAR T cells eradicated the nonirradiated SupT1-CD19/CD22 targets in all donor conditions after 96 h in co-culture in repeated FACS assays (*n* = 2). FACS, fluorescence-activated cell sorting.

We confirmed CAR expression and reporter integration via fluorescence microscopy and quantified transduction efficiency using fluorescence-activated cell sorting (Fig. 1 b). Importantly, expression levels of CAT-CD19-BBZ or 9A8-CD22-BBZ CAR in co-transduced CAR-T cells were comparable with those observed in single-CAR populations, as indicated by the mean fluorescent intensities of the respective reporter genes measured by flow cytometry (Fig. 1 b). Functional validation using cytotoxicity assays against SupT1 and SupT1-CD19/CD22, engineered to express the pan–B cell CD22 and CD19 markers, confirmed that all CAR-T cell populations retained antigen-specific killing capacity (Fig. S1).

To mimic antigen presentation and assess activation under controlled ligand densities, we used glass-supported lipid bilayers (SLBs) reconstituted with CD19, CD22, and ICAM-1 for CAR stimulation or anti-CD3 and anti-CD28 for TCR stimulation (Fig. 1 c). The bilayer composition was identical for all experiments, and ligand density was tuned by uniformly scaling the surface density while maintaining a fixed CD19:CD22 ratio. This platform allows tuning of ligand density to interrogate activation thresholds. We then quantified MAP kinase pathway activation using phospho-ERK (pERK) staining and automated image analysis, identifying each CAR-T population by their fluorescent reporter tags (Fig. 1 d).

pERK signal quantification across three donors (HD1, HD2, and HD3) revealed that dual CAR-T cells consistently activated at lower ligand densities than single CAR-T populations. At 20 molecules/µm^2^ of antigen (i.e., CD19 for anti-CD19 single CARs and dual CARs-T cells and CD22 for anti-CD22 CAR-T cells), ∼20% of dual CAR-T cells were pERK positive, while at this ligand density, single CAR-T cells did not elicit a measurable pERK response above background. At higher ligand densities (>100 mol/µm^2^), however, both dual and single CAR-T cells reached 90% activation, confirming full responsiveness (Fig. 1 e). Based on these results, we selected the higher ligand density condition (>100 mol/μm^2^) for subsequent super-resolution imaging studies of actin remodeling, ensuring maximal activation across all CAR-T subtypes.

To address potential basal signaling in CAR-T cells, we also examined ERK phosphorylation in the absence of specific antigen engagement. Both CAR-T and untransduced T cells were incubated on SLBs functionalized either with ICAM-1 alone or with anti-CD3, anti-CD28, and ICAM-1. Quantification of pERK-positive cells revealed no significant difference between CAR-T and untransduced T cells under any of these two conditions (Fig. 1 f). These results indicate that CAR expression, whether single or dual, does not enhance basal signaling upon ICAM-1 engagement, nor does it alter the proximal TCR signaling response triggered by anti-CD3/anti-CD28 stimulation. Thus, CAR expression does not appear to interfere with canonical TCR-mediated activation in our experimental system.

Together, these findings confirm that CAR-T cells maintain normal basal and TCR-dependent signaling properties. Consequently, the phenotypic and cytoskeletal differences described in subsequent figures can be attributed to antigen-specific CAR signaling rather than nonspecific effects of CAR expression or altered responsiveness to TCR ligation.

### CAR-T cells exhibit disorganized cytoskeletal remodeling with reduced central actin clearance and persistent microvillar projections compared with untransduced T cells

To investigate how CAR engagement affects cytoskeletal remodeling, we used 3D super-resolution STED super-resolution microscopy to visualize F-actin dynamics during IS formation at 1, 3, and 8 min after stimulation. These times points were selected to capture early, intermediate, and mature stages of synapse development. CAR-T cells were activated on SLBs presenting >100 mol/µm^2^ CD19 and CD22 ligands, while untransduced T cells from the same donor were activated on SLBs functionalized with ∼90 molecules/μm^2^ of anti-CD3 (OKT3 clone, K_D_ = 2.6 µM) (Kjer-Nielsen et al., 2004) and anti-CD28, serving as a reference for classical TCR activation.

**Figure 2.**
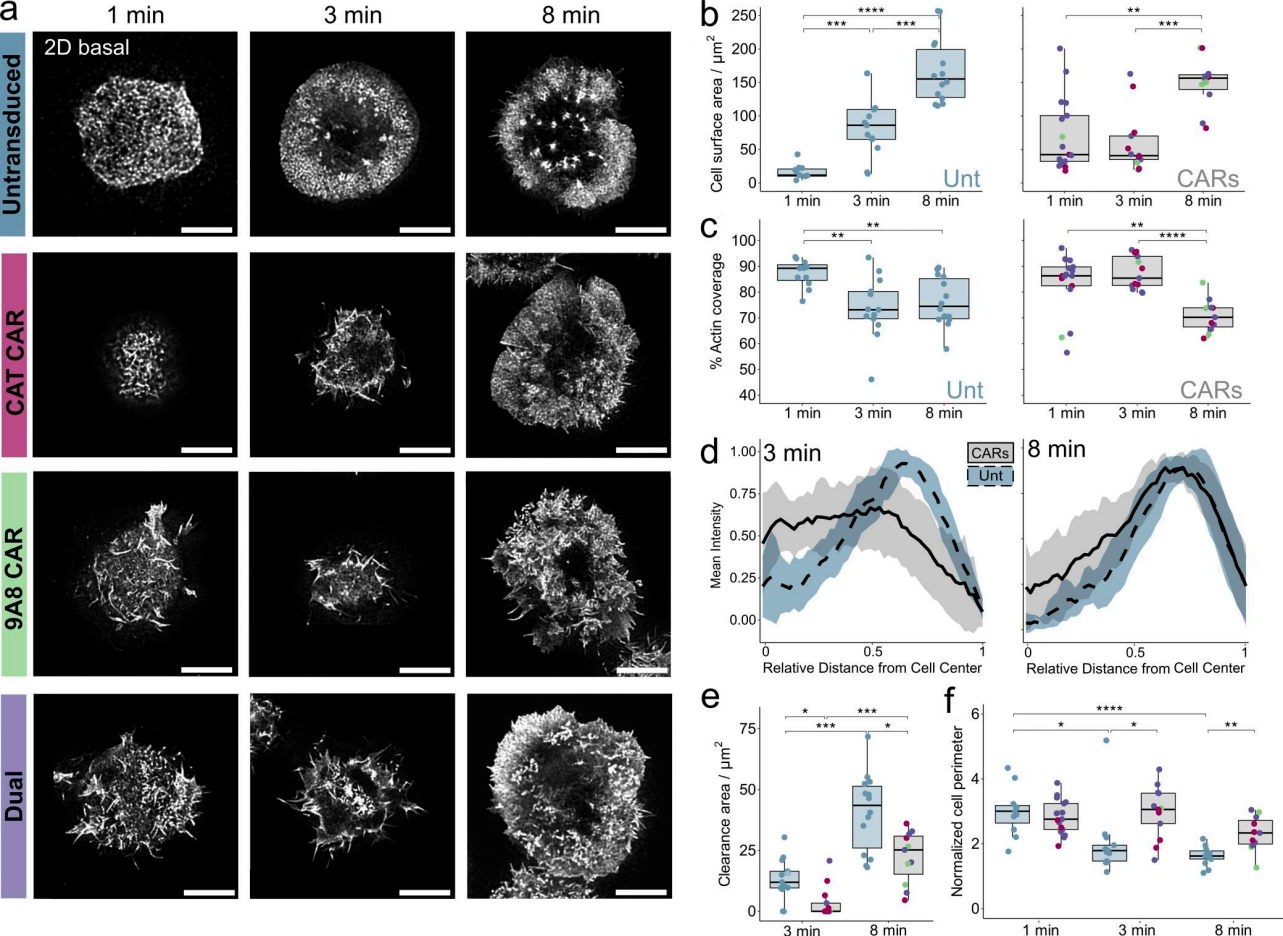
**CAR-T cells exhibit delayed peripheral actin ring formation, reduced central actin clearance, and persistent peripheral protrusions during immune synapse formation.** **(a)** Representative STED images of the 2D basal plane for untransduced primary T cells (blue, top), CAT CAR-T cells (red, upper middle), 9A8 CAR-T cells (green, lower middle), and dual 9A8 and CAT CAR-T cells (purple, bottom) activated for 1 min (left), 3 min (middle), and 8 min (right). Scale bars: 5 µm. **(b)** 2D basal cell surface area (µm^2^) quantification for both untransduced T cells (blue bars, left) and all CAR-T cells (grey bars, right) activated for 1, 3, and 8 min; colors of individual points represent T cell type (blue for untransduced, red for CAT, green for 9A8, and purple for dual CAT and 9A8). **(c)** Percentage of the cell surface area covered by actin across the 2D basal plane for both untransduced (blue bars, left) and CAR-T cells (grey bars, right) for 1-, 3- and 8-min activation; colors of individual points represent T cell type (blue for untransduced, red for CAT, green for 9A8, and purple for dual CAT and 9A8). **(d)** Radial analysis showing the normalized mean fluorescence intensity of F-actin from the center of the cell to the cell edge for untransduced (dashed line) and CAR-T cells (straight line) at both 3- (left) and 8-min (right) activation. Standard deviation of mean intensity is shown with the blue (untransduced) and grey (CARs) bands. **(e)** Quantitative analysis of 3- and 8-min cell synapses showing the clearance area for both untransduced (blue) and CAR-T (grey) cells. **(f)** Quantitative analysis of 1-, 3-, and 8-min cell synapses showing the normalized perimeter for both untransduced (blue) and CAR-T (grey) cells. *n* > 12 CAR-T or untransduced T cells for each activation condition. All box plots show median (center line), interquartile range (IQR; box), and full data range (whiskers). Outliers are defined as values < Q1 −1.5×IQR or > Q3 + 1.5×IQR. For 1-min activation, *n* = 11 untransduced T cells and *n* = 17 CAR-T cells; for 3-min activation, *n* = 13 untransduced T cells and *n* = 13 CAR-T cells; for 8-min activation, *n* = 14 untransduced T cells and *n* = 11 CAR-T cells. In all cases, *n* represents individual cells (technical replicates) pooled from three independent biological donors (*n* = 3 biological replicates). Statistical comparisons were performed using two-tailed nonparametric Mann–Whitney U tests (Wilcoxon rank-sum tests), with Benjamini–Hochberg (BH) correction for multiple comparisons. P values below 0.05 were considered significant using the following notation: *P < 0.05, **P < 0.01, ***P < 0.001, and ****P < 0.0001.

**Figure S2.**
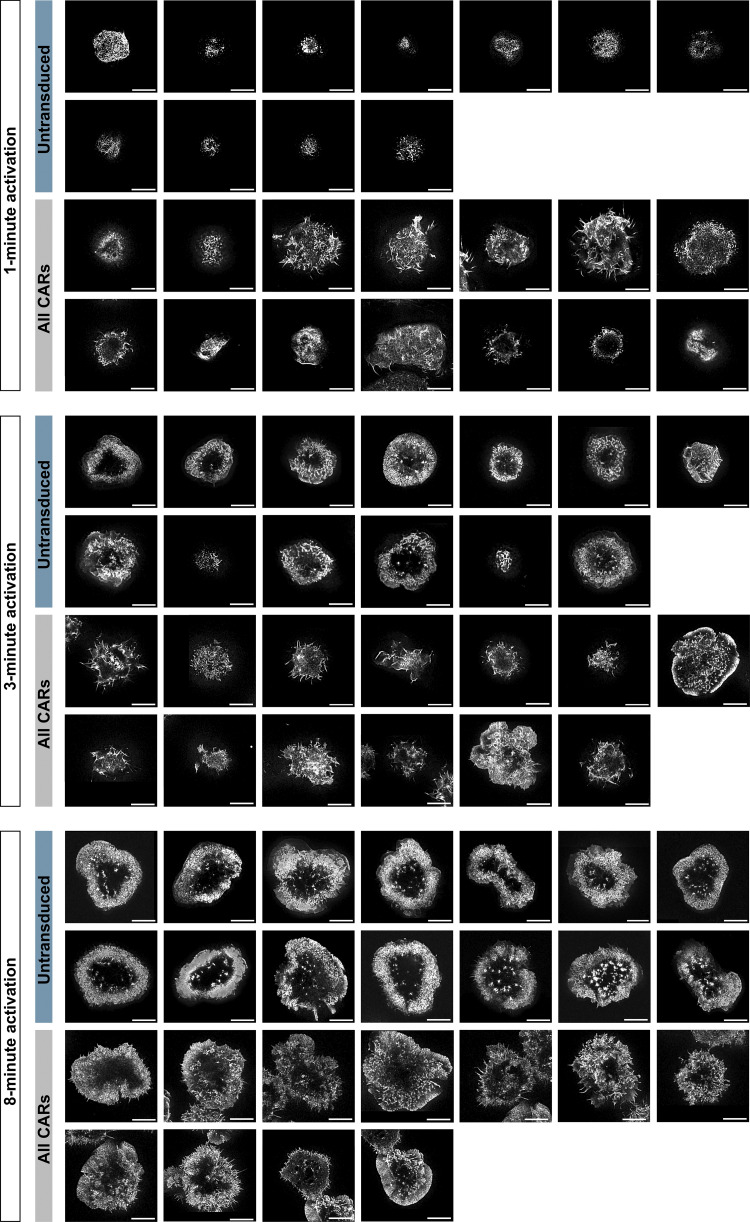
**Full STED dataset of the 2D basal plane for untransduced primary T cells (blue, top), CAT CAR-T cells (red, upper middle), 9A8 CAR-T cells (green, lower middle), and dual 9A8 and CAT CAR-T cells (purple, bottom) stimulated for 1 min, 3 min, and 8 min on the CD19, CD22, and iCAM-1–coated SLB.** Scale bars: 5 µm.

Following stimulation, cells were fixed and stained with SiR-actin, a far-red silicon rhodamine fluorophore conjugated to jasplakinolide, which selectively binds F-actin and is suitable for super-resolution fluorescence imaging. Fig. 2 a shows representative STED images of CAR-T cells and untransduced T cells activated for 1 min (left), 3 min (middle), and 8 min (right) acquired at (75 ± 7) nm lateral resolution as determined by Fourier ring correlation. In all CAR-T subsets, ligand engagement induced cell adhesion, cytoskeleton reorganization, and dynamic cell spreading, albeit with distinct morphological and temporal features compared with untransduced cells (see Fig. S2 for full data sets).

In untransduced T cells, visual examination of STED images revealed a classic progression of actin remodeling. Upon initial contact (at 1 min), actin exhibit a dispersed distribution throughout the cell. However, by 3 min of stimulation, it is possible to discern the emergence of three distinct F-actin zones, which become more structured at 8 min (mature synapse time point): a dense F-actin ring (distal SMAC), concentric actin arcs (peripheral SMAC), and a relatively actin-depleted center (central SMAC)—hallmarks features of a mature IS. In contrast, CAR-T cells exhibited delayed formation of a well-defined peripheral F-actin ring and central clearance region, becoming only discernible at 8 min. Notably, CAR-T cells also exhibited a denser central actin mesh and a smaller actin-depleted region than untransduced T cells.

To quantify these differences, we measured the cell spreading area (Fig. 2 b) and F-actin coverage (Fig. 2 c) relative to total cell area over time. Notably, CAR-T cells expanded from (42 ± 29) µm^2^ to (156 ± 11) µm^2^ between 1 and 8 min. This contrasts with the gradual spreading observed in untransduced T cells: (12 ± 5) µm^2^, (86 ± 22) µm^2^, and (155 ± 36) µm^2^ at 1, 3, and 8 min, respectively. These dynamics were mirrored in actin coverage: untransduced T cells displayed a 20% drop in actin density by 3 min, whereas CAR-T cells only showed a comparable reduction at 8 min. Despite these temporal differences, final spreading area and actin coverage at 8 min were statistically indistinguishable between groups (P = 0.7 and 0.1, respectively).

To further discern differences in actin architecture at the 3- and 8-min time point, we constructed radial fluorescence intensity profiles from the cell center (r = 0) to the cell edge (r = 1). At 3 min, a clear peripheral F-actin ring structure, indicated by a peak at r = ∼0.6, appeared only in untransduced T cells. However, by 8 min, this structure was apparent in both CARs and untransduced T cells (Fig. 2 d). We also assessed actin clearance (Fig. 2 e), finding significantly reduced central clearance in CAR-T cells at both time points (P values = 0.02 for each), indicating delayed formation of a mature central synaptic cleft.

These findings suggest that while CAR-T cells ultimately form actin structures resembling classical synapses, they do so with delayed kinetics and retain a denser central actin mesh. We attribute these differences primarily to CAR-receptor geometry and antigen-binding context. However, we note that our CARs employ a 4-1BB (CD137) costimulatory module, whereas the untransduced T cell reference uses CD28. Although 4-1BB and CD28 signaling differ in pathway utilization and metabolic programming (Kawalekar et al., 2016; Milone et al., 2009), both anti-HER2 CARs with CD28ζ and κ-CARs with either CD28ζ or 4-1BBζ have been reported to exhibit similarly altered actin architectures (Davenport et al., 2018; Xiong et al., 2018). This suggests that the distinct cytoskeletal remodeling observed here likely reflects a shared property of CAR-mediated signaling rather than a specific effect of the costimulatory domain. Indeed, impaired actin clearance from the cSMAC may disrupt granule secretion, as shown in models where PKCδ deletion blocked cytotoxic granule release (Bello-Gamboa et al., 2020). Thus, persistent actin at the synapse center may reduce granzyme B and perforin secretion, compromising cytotoxic function and potentially leading to off-target effects or activation-induced cell death, commonly observed in CAR T cell therapy (Tibbs and Cao, 2022; Bird et al., 2014; Huan et al., 2022).

Furthermore, we also noted qualitative differences in synapse morphology. Untransduced T cells spread smoothly with minimal actin projections from the cell body (i.e., protrusions), while over 50% of CAR-T cells exhibited irregular shapes and thin, finger-like actin projections, visible at all time points (Fig. S2). Quantification of normalized perimeter (Fig. 2 f) revealed that both CAR-T and untransduced T cells exhibited similarly high values at 1 min, consistent with the presence of initial microvilli probing during early antigen contact. In untransduced T cells, the ratio decreased over time (P values ≤0.01, for 1 min compared with both 3 and 8 min), reflecting normal contraction and retraction of microvilli as the synapse matured. In contrast, CAR-T cells retained elevated normalized perimeter at 3 and 8 min, which were significantly higher than untransduced cells (P values = 0.03 and 0.01, respectively; Fig. 2 f), indicating persistent peripheral protrusions. These data suggest that CAR-T cells fail to fully contract and reorganize the actin network, leading to retention of initial microvillar-like structures.

**Figure S3.**
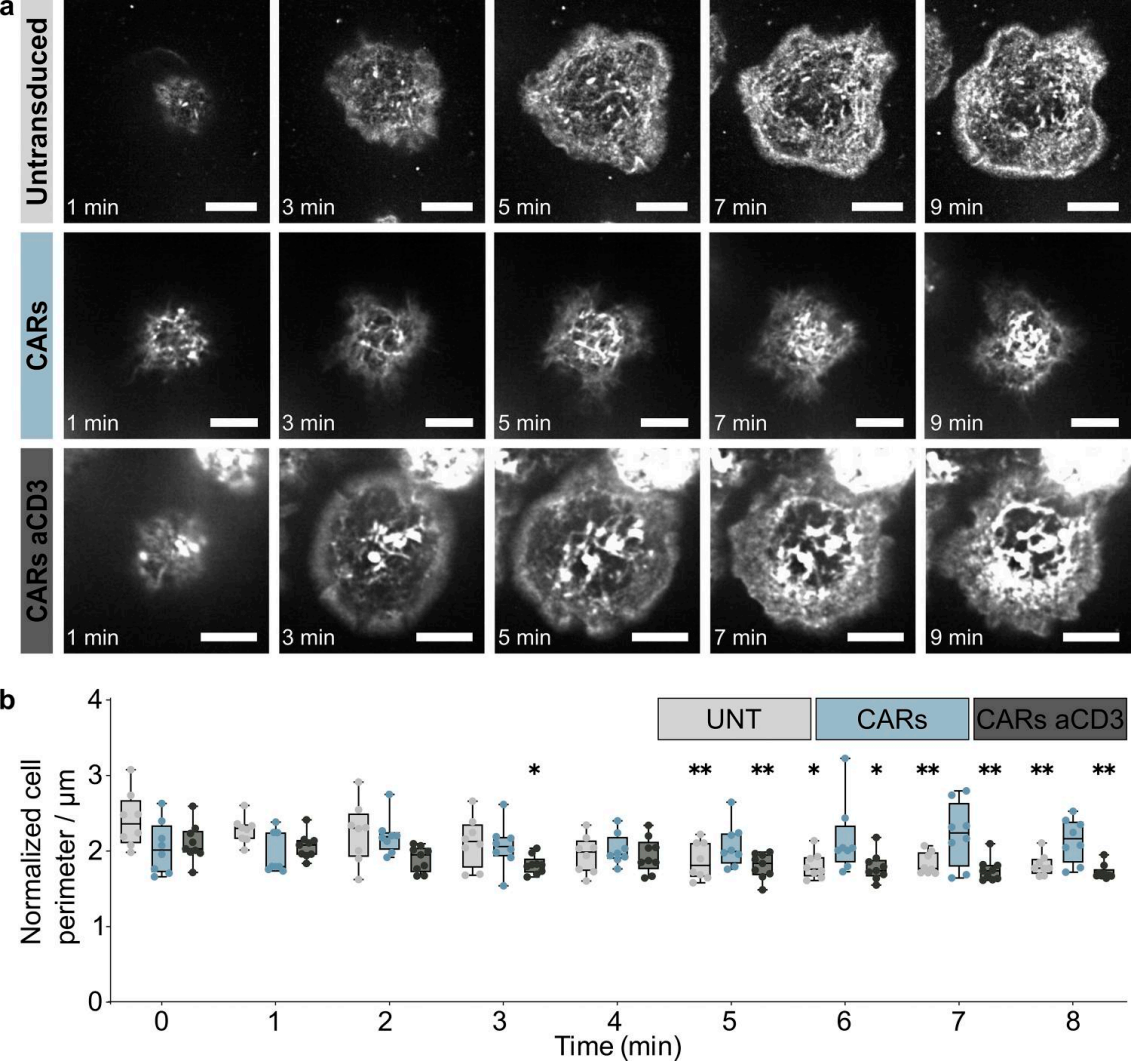
**Live-cell imaging reveals persistent peripheral actin protrusions in antigen-stimulated CAR-T cells, but normal contraction dynamics via TCR stimulation.** **(a)** Representative still frames from live-cell imaging of untransduced Jurkat T cells (blue, top) and CAR-expressing Jurkat T cells (grey, middle and bottom) labeled with SiR-actin and interacting with SLB. SLBs were functionalized with anti-CD3/anti-CD28 and ICAM-1 to stimulate untransduced T cells (top) or CAR-T cells via the endogenous TCR (bottom), or with CD19/CD22 ligands and ICAM-1 to stimulate CAR-T cells via cognate antigen engagement (middle). Images correspond to the basal synapse plane at specific time points following initial contact. Scale bars: 5 µm. **(b)** Quantification of normalized cell perimeter over time for untransduced T cells stimulated with anti-CD3/anti-CD28 and ICAM-1 SLBs (blue), CAR-T cells stimulated via cognate antigen engagement on CD19/CD22- and ICAM-1–functionalized SLBs (grey), and CAR-T cells stimulated via TCR activation on anti-CD3/anti-CD28 and ICAM-1 SLBs (dark grey). The perimeter-to-area ratio reports the temporal dynamics of peripheral actin protrusions during IS formation. Corresponding full time-lapse sequences are shown in Videos 1, 2, and 3. All box plots show median (center line), interquartile range (IQR; box), and full data range (whiskers). For untransduced T cells, *n* = 8 cells; for CAR T cells, *n* = 8 (CD19/CD22- and ICAM-1–functionalized SLBs) and 9 cells (anti-CD3/anti-CD28 and ICAM-1 SLBs). *n* represents individual cells (technical replicates). Statistical comparisons were performed using two-way ANOVA followed by Dunnett’s multiple comparisons test. P values below 0.05 (relative to 1-min interval) were considered significant using the following notation *P < 0.05, **P < 0.01.

To complement these static snapshots, we performed live-cell imaging of Jurkat CAR-T and untransduced Jurkat T cells labelled with SiR-actin to visualize F-actin dynamics during engagement with activating SLBs (Fig. S3; and Videos 1, 2, and 3). Imaging was performed on a spinning disk super-resolution microscope with optical photon reassignment (SoRa), achieving a lateral resolution of 230 ± 20 nm as determined by Fourier ring correlation. Time-resolved analysis confirmed that untransduced T cells exhibited a gradual decrease in cell perimeter-to-area ratio, with significant reductions beginning at 5 min (P values ≤0.011 thereafter, all compared with 1 min), consistent with microvilli retraction and normal synapse maturation (Fig. S3 and Video 1). In contrast, CAR-T cells did not exhibit a significant reduction in perimeter-to-area ratio at any time point within the imaging window (all P values >0.19 compared with 1 min), indicating sustained peripheral actin protrusions and failure to undergo normal synapse contraction (Fig. S3 and Video 2). Importantly, to determine whether this phenotype reflects antigen-specific CAR signaling rather than an intrinsic property of CAR expression, we repeated the live-cell imaging experiments of Jurkat CAR-T cells using anti-CD3/anti-CD28 stimulation. Under TCR-mediated activation, CAR-T cells exhibited normal contraction dynamics, with significant reductions in perimeter-to-area ratio beginning at 3 min (P = 0.012; P ≤0.022 after 5 min, all compared with 1 min), and formation of a stable synapse (Fig. S3 and Video 3). Thus, the persistent microvillar projections and aberrant cytoskeletal remodeling observed upon engagement of the CAR by its cognate antigen are specifically driven by antigen-dependent CAR signaling and are not elicited by canonical TCR activation in CAR-expressing cells.

**Video 1.** **F-actin live dynamics in untransduced Jurkat T cells**. Time-lapse spinning disk confocal super-resolution microscopy of SiR-actin–labeled untransduced T cells engaging with anti-CD3/anti-CD28 and ICAM-1 SLBs. Basal synapse plane shown. Images were acquired every 10 s and displayed at 7 fps. Scale bar: 5 µm. Related stills and quantification are shown in Fig. S3.

**Video 2.** **F-actin live dynamics in CAR-expressing Jurkat T cells stimulated via cognate antigen.** Time-lapse spinning disk confocal super-resolution microscopy of SiR-actin–labeled CAR-T cells engaging with CD19/CD22 and ICAM-1 SLBs. Basal synapse plane shown. Images were acquired every 10 s and displayed at 7 fps. Scale bar: 5 µm. Related stills and quantification are shown in Fig. S3.

**Video 3.** **F-actin live dynamics in CAR-expressing Jurkat T cells stimulated via TCR.** Time-lapse spinning disk confocal super-resolution microscopy of SiR-actin–labeled CAR-T cells engaging with anti-CD3/anti-CD28 and ICAM-1 SLBs. Basal synapse plane shown. Images were acquired every 10 s and displayed at 7 fps. Scale bar: 5 µm. Related stills and quantification are shown in Fig. S3.

Together, the live-cell and fixed-cell data indicate that CAR-T cells activated through cognate antigen engagement retain initial microvillar structures and fail to complete the normal spreading–contraction cycle, resulting in a disorganized or partially matured IS architecture.

### Actin remodeling during mature synapses in CAR-T cells features persistent microvilli, reduced F-actin foci, and enlarged actin mesh structures indicative of incomplete cytoskeletal contraction

**Figure 3.**
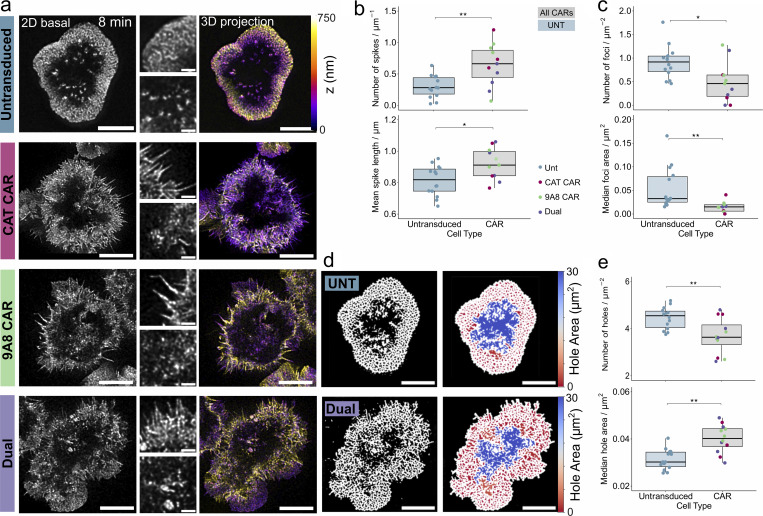
**Mature CAR-T cell synapses retain more numerous microvilli, exhibit reduced F-actin foci, and display enlarged actin mesh holes. (a)** Representative STED images of the actin cytoskeleton after 8 min of activation for untransduced and CAR-T cells. Showing the full cell (first panel), zoomed in regions of the cell perimeter (top, middle panel) and cell center (bottom, middle panel) at the synapse, and reconstructed 3D projections of the image stack taken at different axial positions (step size of 150 nm up to 750 nm). Scale bars: 5 µm for full cells and 1 µm for zoom regions. **(b)** Perimeter analysis of untransduced (blue) and CAR-T cells (grey) at 8-min synapse, quantifying the number of spikes per cell in µm^−2^ (top) and mean spike length in µm (bottom). Colors of individual points represent T cell type (blue for untransduced, red for CAT, green for 9A8, and purple for dual CAT and 9A8). **(c)** Quantification of actin foci at the actin clearance center of the cells across both untransduced (blue) and CAR-T cells (grey), measuring density of foci at the cell clearance area in µm^−2^ (top), and median area of foci clusters in µm^2^ (bottom). **(d)** Segmentation of the actin mesh network, including cell mask (left) and color-coded segmentation map (right) for an example untransduced (top) and dual CAR-T cell (bottom). Color bar shows area of holes in µm^2^. Scale bars: 5 µm. **(e)** Analysis of the actin mesh from segmentation, comparing the mesh density of both untransduced and CAR-T cells at 8-min synapse through number of holes per µm^2^ (top) and median hole area in µm^2^ (bottom). All box plots show median (center line), interquartile range (IQR; box), and full data range (whiskers). Outliers are defined as values < Q1 −1.5×IQR or > Q3 + 1.5×IQR. For untransduced T cells, *n* = 14 cells, for CAR T cells, *n* = 11 cells. *n* represents individual cells (technical replicates) pooled from three independent biological donors (*n* = 3 biological replicates). Statistical tests performed were two-tailed nonparametric Mann–Whitney U tests (Wilcoxon rank-sum tests). P values below 0.05 were considered significant using the following notation: *P < 0.05, and **P < 0.01.

To further investigate the differences in actin remodeling during mature ISs, we reconstructed 3D projections from axial STED image stacks (acquired at 150 nm steps up to 750 nm) of the actin cytoskeleton in both CAR-T and untransduced T cells at the 8-min time point (Fig. 3 a).These reconstructions enabled the distinction between cytoskeleton structures at the basal membrane and those protruding above it.

Both single and dual CAR-T cells exhibit prominent rod-like protrusions extending above the basal plane, with average length of 0.91 ± 0.08 µm. Quantitative analysis (Fig. 3 b) showed that CAR-T cells had both significantly more and longer microvilli compared with untransduced T cells (mean number: 0.7 ± 0.2 µm^−1^ vs. 0.3 ± 0.1 µm^−1^; mean length: 0.91 ± 0.08 μm vs. 0.82 ± 0.07 µm; P values = 0.009 and 0.02, respectively), consistent with our live-actin imaging results but now resolved and quantified at higher spatial resolution. These actin-rich, finger-like protrusions indeed correspond to microvilli, which serve as dynamic environmental sensors on the T cell surface, acting as primary sites for scanning and local receptor signaling (Jung et al., 2016). In native T cells, microvilli are abundant during early contact but retract as the synapse matures, allowing actin rearrangement and central actin clearance to occur. However, our observations show that CAR-T cells preferentially retain microvilli compared with untransduced cells even at intermediate (3 min) and mature (8 min) stages, consistent with the delayed or incomplete actin contraction. Supporting this, recent lattice light-sheet and total internal reflection fluorescence imaging studies have reported hyperstabilized microvillar contacts in CAR-T cells (Beppler et al., 2023), likely due to increased CAR affinity or elevated antigen density on cancer cells. The persistent microvilli dynamics may alter antigen scanning and signal integration, prolonging contact duration while potentially impairing signaling efficiency and increasing susceptibility to exhaustion.

In parallel, we observed notable differences in F-actin organization. Untransduced T cells exhibited distinct, centralized F-actin–rich clusters—commonly referred to as F-actin “foci”—at the basal plane. These structures typically colocalize with TCR signaling microclusters and tyrosine-phosphorylated intermediates (Kumari et al., 2015). In contrast, CAR-T cells showed significantly fewer and smaller actin foci (Fig. 3 c; P value = 0.02 and 0.001, respectively), suggesting impaired actin-dependent signal amplification. Given that WASP, a key regulator of actin foci, is essential for TCR-mediated signaling and mechanotransduction (Mandal et al., 2023), the loss of these structures in CAR-T cells may weaken synapse stability and reduce signal transduction. This may explain the previously reported shorter duration of CAR-T synapses and premature detachment from target cells, as observed by Davenport et al. (2018).

**Figure S4.**
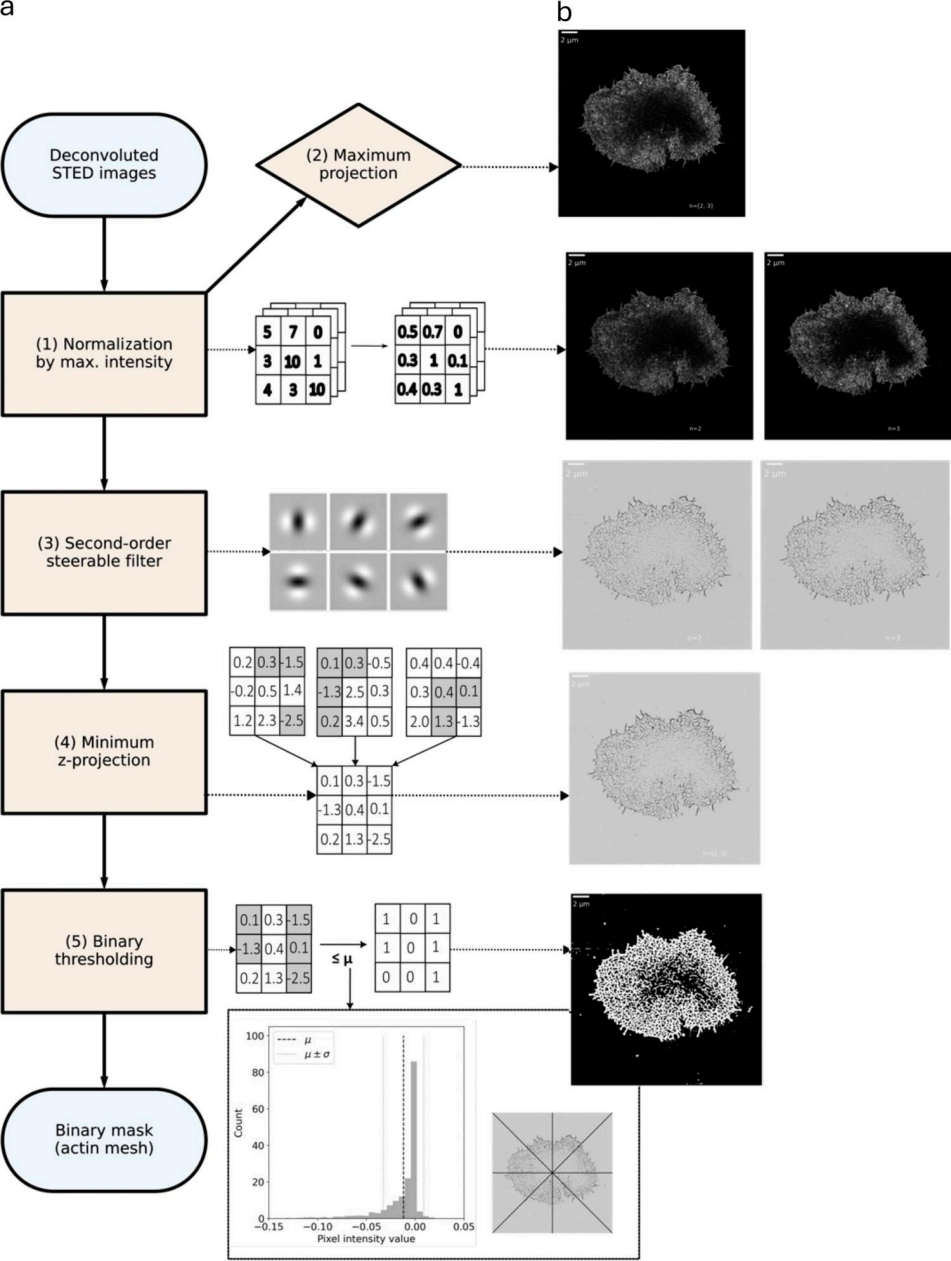
**Overview of the main steps comprising the mesh segmentation pipeline in ActinMeshure package. (a)** Flow chart: each step is accompanied by a schematic representation of the mathematical manipulation (linked with an arrow). Black box shows how the dynamic threshold algorithm samples data from the original image to obtain the threshold value “μ.” **(b)** Example output: each step of the segmentation process is visualized with an example of the output for the basal actin meshwork of one CAR-T cell with 8-min activation time. Scale bars = 2 μm.

To further assess cortical actin architecture, we developed a streamlined image segmentation (Fig. 3 d, left) and mesh quantification (Fig. 3 d, right) pipeline for analysis of the STED datasets (Fig. S4). Analysis of the basal actin mesh revealed a significantly larger median mesh hole size in CAR-T compared with untransduced T cells (40 ± 4 nm vs. 30 ± 3 nm; P value = 0.001) (Fig. 3 e, top). A dense and well-organized actin network is critical for maintaining stable physical contact between a T cell and its target. Thus, the observed enlarged mesh holes in CAR-T cells may contribute to previously reported premature detachment events. Conversely, untransduced T cells displayed higher mesh hole density (4.5 ± 0.3 vs. 3.6 ± 0.4; P value = 0.006) (Fig. 3 e, bottom), suggesting a denser and more tightly woven actin network. In CAR-T cells, this reduced mesh density may hinder efficient actin polymerization and lead to suboptimal organization of the IS. Such structural disorganization could impair the recruitment and clustering of signaling molecules essential for robust and sustained T cell activation.

Together, these findings define a distinct actin remodeling program in CAR-T cells during mature synapse formation—characterized by persistent microvilli, sparse and small F-actin foci, and a less dense actin mesh—reflecting incomplete cytoskeletal contraction and partial synapse maturation.

In summary, actin remodeling in CAR-T cells proceeds in a disorganized and incomplete manner, with less prominent large-scale cytoskeleton reorganizations, persistent peripheral microvilli, reduced actin clearance, and reduced actin foci structures, features indicative of partial synapse maturation. We note that, although we distinguished single- and dual-CAR–expressing cells throughout our analyses, their cytoskeletal remodeling dynamics and synapse morphologies were largely comparable. We therefore conclude that the principal cytoskeletal features described here are generalizable across single– and dual–CAR-T populations. These alterations may disrupt signal transduction and granzyme B secretion, ultimately compromising their cytotoxic function and reducing tumor cell killing efficiency. Persistent microvilli retention in CAR-T cells may also alter scanning and signaling, potentially accelerating T cell exhaustion. Additionally, the sparser actin meshwork and enlarged holes undermine synapse integrity, potentially promoting premature detachment and diminishing immune efficacy. Understanding these distinct patterns of actin remodeling between CAR-T cells and untransduced T cells is essential for developing strategies to enhance actin clearance and optimize cytoskeletal dynamics, ultimately improving CAR-T cell efficacy and therapeutic outcomes.

## Materials and methods

### CAR-T cell preparation

The CAT-CD19-BBZ and 9A8-CD22-BBZ CAR constructs, comprising an scFv (CAT or 9A8), HuCD8α hinge and transmembrane domains, 4-1BB costimulatory domain, and CD3ζ signaling domain, were provided by Autolus. Due to proprietary restrictions, full nucleotide sequences of CAR constructs are not publicly available; key structural features are described by Ghorashian et al. (2019) and Kokalaki et al. (2023).

PBMCs were isolated by Ficoll density centrifugation of blood obtained from healthy donors. Use of human cells in this study was approved by the NHS Health Research Authority Research Ethics Committee (reference 16/LO/1290), and oral informed consent was obtained from all participants.

Freshly isolated PBMCs were cultured in TexMACS medium (130-097-196; Miltenyi Biotec) and activated with CTS Dynabeads CD3/CD28 (40203D; Thermo Fisher Scientific) at a 1:3 lymphocyte-to-bead ratio in MACS GMP Cell Differentiation Bags (170-076-405; Miltenyi Biotec). Cells were incubated at 37°C with 5% CO_2_, and beads were magnetically removed on day 5, followed by a 48-h rest before antigen stimulation. For lentiviral transduction, overnight bead-activated T cells (0.5 × 10^6^) were suspended in TexMACS and transduced with a lentiviral vector encoding CD19 CAR (CAT) and CD22 CAR (9A8) using RetroNectin-coated 24-well plates with 1 ml of LV supernatant per well. Spinoculation was performed at 1,000 *g* for 40 min at room temperature. Typically, 2–10 × 10^6^ activated T cells per donor per construct were seeded for transduction. Successfully transduced primary T cells were expanded and used for STED imaging of fixed synapses and functional assays.

SupT1 (95013123; ECACC) cell lines were provided and engineered to express the CD19 or CD22 or both antigens at various levels by Autolus as described by Kokalaki et al. (2023).

For live-cell imaging experiments, Jurkat E6.1 T cells (TIB-152; ATCC) were transduced with the same CAR constructs to generate CAR-expressing cell lines. Lentiviral transduction was performed using RetroNectin-coated 24-well plates with 1 ml of lentiviral supernatant per well, followed by spinoculation at 1,000 × *g* for 40 min at room temperature. Transduced cells were cultured in RPMI-1640 supplemented with 10% FBS and 1% penicillin-streptomycin at 37°C with 5% CO_2_.

### Lipid preparation

In brief, to prepare the lipid stock solutions for the SLB, 97.4% DOPC (850375C; Avanti Polar Lipids), 2% DGS-NTA(Ni) (790404C; Avanti Polar Lipids), 0.1% Biotinyl-Cap-PE (870273C; Avanti Polar Lipids), and 0.5% PEG5,000-PE (880220P-200 MG; Merck) molar percentages were mixed in chloroform in a round bottom flask and vortexed. This mixture was dried in a fume hood under a flow of nitrogen while constantly rotating the flask. To ensure complete removal of the chloroform, the lipids were placed in a vacuum oven at room temperature for 2 h. Following this, the lipid mixture was rehydrated in lipid buffer (0.1% BSA, 2 mM MgCl_2_, and 1 mM CaCl_2_ in PBS) and vortexed to form a 4 mM solution. This mixture was extruded 21 times through a 100-nm pore-size polycarbonate filter to form unilamellar vesicles. The lipid solution was then diluted to 0.4 mM and aliquoted into 1-ml tubes to be stored under argon at 4 °C until required. The size and polydispersity of the vesicles in solution was measured using dynamic light scattering with Malvern Zetasizer Ultra-Red at 25°C. The data were analyzed using ZS XPLORER 2.1.0.15, where the average size was recorded with an average diameter for the vesicles of 143.6 ± 3.0 nm with a polydispersity of 0.062.

### Preparation and antigen functionalization of glass SLBs

Glass coverslips were cleaned by 30-min sonication in 2% Hellmanex detergent and then sequentially washed with deionized water and with ethanol (80%) before drying then under a flow of nitrogen. These glass coverslips were plasma cleaned (Diener Zepto, 40 kHz generator, 90 s at 70W power) and immediately attached to a 6-chamber sticky-slide (sticky-Slide VI 0.4, 80608; Ibidi). Then, 50 μl of the 0.4 mM lipid vesicles were added to each chamber and incubated at room temperature for 20 min to allow the formation of the glass SLBs. Chambers were then washed with 200 μl of lipid PBS buffer solution (0.1% BSA + 2 mM MgCl_2_ + 1 mM CaCl_2_) for a total three washes. Each chamber was then loaded with 100 μl of blocking buffer (0.1 mM NiCl_2_ in PBS with 2% BSA) and incubated at room temperature for 20 min. Following three washes with the lipid buffer solution, the SLBs were functionalized. First, 100 μl of 12.5 µg/ml streptavidin (228-11469-2; Cambridge Bioscience RayBiotech) solution prepared in lipid buffer were loaded in each chamber for 20 min. Following three washes with lipid buffer, 100 μl of an antigen mixture were added to the SLBs at room temperature for 30 min. For the anti-CD3 and anti-CD28 functionalized SLBs, this mixture was comprised of 200 ng/ml his-tagged ICAM-1 (A42524; Thermo Fisher Scientific), 1.69 µg/ml biotinylated anti-CD3 (317320; BioLegend), and 1.69 µg/ml biotinylated anti-CD28 (302904; BioLegend). For the ICAM-1 only functionalized SLBs, only 200 ng/ml of his-tagged ICAM-1 was used. For the CD19 and CD22 functionalized SLBs, this mixture was comprised of 200 ng/ml his-tagged ICAM-1, biotinylated and his-tagged CD19 (CD9-H82E9-25ug; Acrobio), and CD22 (11958-H08H-B; Sino Biological). For pERK experiments, concentrations of CD19 and CD22 ranged from 0.002 – 3.00 µg/ml and 0.006–6.83 µg/ml, respectively. For STED actin imaging, the concentration of CD19 and CD22 added were 1.60 µg/ml and 3.64 µg/ml respectively. Finally, the bilayers were washed three times with lipid buffer solution and moved to a humidified chamber at 37 °C at 5% CO_2_ until further use. Lateral mobility of proteins in the SLBs was examined via FRAP determining that 90% of proteins diffused freely with a diffusion coefficient of 0.2 µm^2^ s^−1^ at 37°C.

### FRAP

To test the mobility of the lipid bilayer, we used FRAP on an Olympus FluoView FV1200 Confocal Laser Scanning Microscope fitted with a 60× oil PlanApo N objective with NA 1.40. For FRAP, the bilayer was prepared as above but functionalized with AlexaFluor488-conjugated streptavidin (S32354; Thermo Fisher Scientific). Images were acquired in a square region of 46 × 46 µm using a 488 nm laser at 2% power for 4 frames. Then, a small area of <3 µm^2^ was bleached using the same laser at 50% power for 20 s. Following this, the full field of view was imaged for a further 96 frames again using 2% power to record the fluorescence recovery. The fluorescence recovery data were processed using FIJI and quantified using the Bessel function by Soumpasis (1983), resulting in a diffusion coefficient of 0.2 µm^2^/s.

### Flow cytometry analysis of protein density on the lipid bilayer

To calibrate the density of proteins, the lipid bilayer was formed onto blank silica beads before being functionalized with proteins for detection using flow cytometry. All steps were performed at RT. Briefly, 130 μl of blank silica beads (SS05003-BAN-0.5g; Stratech) were washed with 3 × 1 ml water and 3 × 1 ml PBS, followed by the addition of 1 ml of the lipid stock (see Materials and methods section for preparation). This was shaken at 1,000 g for 20 min. The beads were then washed with 3 × 1 ml LB. As in the bilayer preparation above, 0.1 mM NiCl_2_, in 2% BSA, was then added to the beads for 20 min, shaking at 1,000 *g*.

For the streptavidin calibration, 1 μl of the bilayer coated beads was added to 26 wells of a 96-well plate. Two of these wells were controls and simply resuspended in LB. The remaining wells were functionalized with varying dilutions of streptavidin-AlexaFluor488 (S32354; Thermo Fisher Scientific). The initial concentration of streptavidin was 50 µg/ml in lipid buffer, followed by dilutions in LB. Each streptavidin concentration was added to separate wells incubated in the dark for 20 min. Following this, the plate was washed three times with LB. Each well was then resuspended in LB and stored in the 96-well plate at 4°C until needed.

For the CD19 and CD22 calibration, the beads were prepared as above, but before adding to the 96-well plate, 1 ml of 12.5 µg/ml unfunctionalized streptavidin (228-11469-2; Cambridge Bioscience RayBiotech) in LB was added for 20 min. The beads were washed three times in LB and then added to 24 wells. The initial concentration of CD19 and CD22 used was and 12.5 µg/ml, respectively, both in 200 ng/ml ICAM-1 in LB, followed by serial dilutions. Each ligand concentration was added to a separate well and incubated for 20 min before washing three times with LB. Both CD19 and CD22 were added together to the bilayer as in later imaging experiments. AlexaFluor488 fluorescently labelled aCD19 or aCD22 (1:200, FAB1968G-100UG; Bio-Techne) was added to the desired wells and incubated in the dark for 1 h before washing three times in LB and stored at 4°C until needed.

CytoFLEX S (Beckman Coulter) at the Zayed Centre for Research was used to measure the fluorescence intensity of the beads. Calibration beads were used to quantify the data (BLI488A-1; Stratech). Two repeats were measured for each condition, each recording 10,000 events. FlowJo (v.10.8.2) was used to process the bead data. In summary, the beads were gated for singlet populations (FSC-H vs. FSC-W). From this population, the mean AlexaFluor488 area was recorded. Using the gradient obtained from the calibration beads, the final density of each protein on the bilayer was quantified.

### CAR-T cells and primary T cell activation on SLBs

Cell populations consisted of an untransduced population as well as a mixed CAR-T product containing: single-CAT-CD19-CAR, single 9A8-CD22-CAR, and dual CAT-CD19/9A8-CD22-CARs.

For fixed imaging experiments, cells were centrifuged at 300 *g* for 5 min and resuspended in PBS (Gibco) in a final concentration of around 5 × 10^6^ cells/ml. Cells were gently mixed, and 100 μl was loaded to the corresponding chamber slides with the functionalized SLBs to activate the CAR products. The chamber slide was transferred inside the incubator at 37°C for 1, 3, or 8 min. At the end of the activation time, the liquid in all the chamber slides was then replaced by 100 μl of pre-warmed 7% PFA (LifeTech) in PBS and incubated for 30 min at room temperature.

For live-imaging experiments, 5 × 10^5^ cells were incubated overnight at 37°C and 5 % CO_2_ in 2 ml of cell cultured media containing 200 nM Silicon Rhodamine (SiR, SC001; Spirochrome) actin live-cell dye. Next day, cells were washed by spinning them down at 140 RCF for 7 min and resuspended in live-cell imaging buffer (A59688DJ; Thermo Fisher scientific) to a final concentration of 2 × 10^6^ cells/ml.

### Immunostaining

The fixation solution was removed, and chamber slides were washed three times with 60 mM glycine in PBS. Fixed cells were permeabilized by adding 100 μl of 0.1% Triton X-100 for 5 min and then washed three times with 60 mM glycine in PBS. 100 μl of blocking buffer (5% BSA in PBS) was added to each chamber and incubated for 60 min in the dark.

For the confocal pERK imaging, the blocking solution was then replaced with 100 μl of the pERK antibody (9101S; Cell Signaling, Rabbit) at 1:500 dilution in 5% BSA in PBS and left to incubate at 4°C overnight. The slide was washed three times for 3 min with 5% BSA in PBS before incubating with AlexaFluor647 fluorescently labelled anti-Rabbit FAB (A21246; Thermo Fisher scientific) diluted 1:1,000 in 5% BSA in PBS for 1 h at room temperature. The slide was washed three times with PBS, and 100 μl of 300 nM DAPI in PBS was added to the cells for 5 min. The slide was washed three times with PBS and ready to image for pERK experiments.

For the STED actin imaging experiments, the blocking solution was then replaced with 100 μl of 2 µM SiR actin (SC001; Spirochrome) in PBS for 15 min at room temperature. The slide was washed three times with PBS and left in cytoskeleton buffer (50 mM imidazole, 50 mM KCl, 0.5 mM MgCl_2_, 0.1 mM EDTA, and 1 mM EGTA at pH 6.8).

### Confocal pERK imaging

Images were taken with the Leica LAS AF software using a Leica SP5 confocal microscope fitted with a 40× oil HCX PL APO objective with NA 1.25. DAPI was imaged with the UV laser at 5% power, the GFP with the 488 nm laser at 11% power, the mCherry with the 561 nm laser at 20%, and the pERK via AlexaFluor647 with the 633 nm laser at 25% power. Images were taken at room temperature.

### STED actin imaging

Images were taken with the Leica LAS X software using a Leica TCS SP8 STED 3X fitted with a 100× HC PL APO CS2 oil objective with NA 1.4. To image SiR actin, the 633 nm laser was used at 10% power with the STED 775 nm laser at 30% power. Line and frame average was 4 and 1, respectively, with line and frame accumulation at 1 and 3, respectively. Cells were identified as GFP and/or mCherry positive using the WLL at 21% power for 488 nm laser and the 6% power at 561 nm, respectively. Images were taken at room temperature. The resulting images were deconvoluted using Huygen’s Professional’s software (version 22.04.0p5). For display, representative single-cell images were cropped from the deconvolved datasets and padded with a black background where necessary to standardize panel dimensions.

### SoRa live actin imaging

Live-cell F-actin dynamics were imaged using a dual-camera (ORCA-Fusion BT Digital CMOS) spinning disk SoRa (CSU-W1 SoRa, Nikon) and a 60× objective (1.49 NA) at 37°C and 5% CO_2_. Prior to imaging, the SLB–functionalized microscope slide was equilibrated on the sample stage for 10 min to reach 37°C. Jurkat CAR-T or untransduced T cells (100 μl) were then added to the imaging chamber at a concentration of 2 × 10^6^ cells/ml, and acquisition was initiated immediately to capture initial cell settling and activation on the functionalized surface. Images were acquired with Nikon NIS-Elements every 10 s for 20 min using a 638 nm laser at 30% power with a 708/75 nm bandpass emission filter. A 2.8× SoRa magnification was applied, yielding a final pixel size of 78 nm for high-resolution F-actin visualization.

After acquisition, all images were deconvoluted using the Richardson-Lucy 2D method with a maximum of 20 iterations and an effective pinhole size of 0.01 in the Nikon NIS-Elements software.

### Image analysis

Radial, cell and clearance area, percentage actin coverage, and foci quantification analysis were all performed on the basal plane only.

#### Radial analysis

Radial analysis was performed in Fiji (v.2.14.0) with the “radial profile” plugin used to measure intensity of actin values as the radius increased from the cell center to the cell edge. This intensity was normalized for each cell, scaling maximum intensity to arbitrary “1,” and minimum intensity to “0,” as well as giving a relative radius of 0–1 from the cell center to the outermost point at the cell edge.

#### Clearance area quantification

Fiji (v.2.14.0) was used to calculate the clearance area of the cells. For this, the images were first inverted, and a threshold was then set to only detect the background of the image, not the cell. “Analyze particles” was then used to measure the size of the clearance area at the center of the cell, including any foci.

#### Cell area, perimeter, and percentage actin coverage quantification

Fiji (v.2.14.0) was used to calculate the percentage actin coverage cells. For this, a threshold was first set to detect the actin staining in the cell. Then, Analyze Particles including holes was used to measure the full cell area (A) and perimeter (P). This was repeated but excluding holes to measure only the actin coverage at the basal surface. The percentage actin coverage was calculated using these values. The normalized perimeter (Pnorm) was also calculated using these values and the following equation:

$$P_{norm}=\frac{P}{2\sqrt{\pi A}}.$$

#### Microvilli quantification

Microvilli quantification was performed using “CellGeo” on MATLAB v. 2014 (Tsygankov et al., 2014). First, a mask of each image was created using the “MovThresh,” which was segmented in “BisectoGraph,” This allowed us to use “FiloTracker” to quantify the total number and mean length of microvilli per cell. Parameters for microvilli detection used were both 20 pixels (c. 0.6 μ m) for critical width and length.

#### FOCI quantification

The clusters of potential actin foci within the actin clearance area were quantified using Fiji (v.2.14.0). The clearance area ROI from the inverted image, as defined above, was applied to the standard images, and Analyze Particles was used to measure any actin clusters within the clearance area. This did not include any actin still connected to the outer actin mesh, which is more likely to represent a lack of actin clearance and is not observed as well-defined clusters of actin. Through Analyze Particles, the median area of actin clusters per cell and the density of actin clusters within each cell’s clearance area were measured.

#### Segmentation analysis

The actin mesh segmentation algorithm was based on the work of Fritzsche et al. (2017). Here, this method was adapted and streamlined into Python as a standalone library of several modules built to segment and quantify the actin mesh. The library also supports batch processing of STED data. More details, which are specific to the software implementation, can be found in the documentation that supplements the installable “ActinMeshure” package on the GitHub of the Simoncelli Lab.

First, the actin mesh was segmented to delineate the mesh from the background. The mesh segmentation pipeline shown in Fig. S3 was applied to the basal frames (the first three bottom-most frames which are closer to the focal plane, <0.10 µm). In brief, each frame in the stack was normalized by the maximum pixel intensity. To highlight the mesh structure qualitatively from the respective selected frames, a maximum z-projection was applied to the sub-stack of frames. At every pixel position, the maximum value across all frames was retained. A ridge detection algorithm was applied to the respective sub-stack of the normalized data. To highlight the mesh outline, a second-order steerable Gaussian filter was applied (σ = 2) in 21 orientations in the range [0, 180] degrees. This was a compromise between sampling enough orientations and computational performance. The minimum projection of the 21 responses was taken for every frame analyzed. A minimum z-projection was then applied to all frames of interest. At this stage, the mesh was highlighted in a single image. A dynamic binary thresholding algorithm was then applied to segment the mesh. A sample of four lines (the two diagonals and the two orthogonal central lines along the two axes) was aggregated, and the mean was taken as the unique threshold for every image. Values less than the threshold were labelled as foreground (1), and the rest were labelled as background (0). Resulting in the entire actin mesh being delineated with minimal noise.

#### Mesh quantification

After segmenting the actin mesh structure from the deconvoluted STED data as above, the binary data were used to extract more quantitative information from the STED images. Specifically to measure the median hole size and number of holes in the actin mesh, the resulting binary mask was inverted, and both were quantified using the measure.regionprops module of the scikit-image library.

### Statistical tests

Statistical tests were carried out in R (v. 4.3.1) using the rstatix package. Unless otherwise stated, experiments were performed using cells derived from three independent biological donors (*n* = 3 biological replicates). Individual data points in figures represent single cells (technical replicates) pooled across donors. For comparing two conditions, two-tailed nonparametric Wilcoxon rank-sum (Mann–Whitney U) tests were used, while for comparing more than two conditions, two-tailed nonparametric Wilcoxon signed-rank tests with Benjamini–Hochberg correction for multiple hypothesis testing were used. Because nonparametric tests were used, no assumptions of normality were required, and data distributions were not formally tested for normality. For time-course analyses involving multiple experimental conditions and time points, two-way ANOVA followed by Dunnett’s multiple comparisons test was used, as specified in the corresponding figure legends. P values below 0.05 were considered significant using the following notation: *P < 0.05, **P < 0.01, ***P < 0.001, and ****P < 0.0001.

### Online supplemental material

Fig. S1 shows a co-culture assay confirming cytotoxic ability of dual CAR-T cell products from PBMCs. Fig. S2 shows the full STED dataset of CAR T cells and untransduced T cells at 1, 3, and 8 min of activation. Fig. S3 shows live-cell F-actin dynamics in Jurkat CAR-T and untransduced T cells, with quantification of cell perimeter-to-area over time. Fig. S4 shows the segmentation pipeline of the actin mesh analysis. Video 1 shows live-cell F-actin dynamics in untransduced Jurkat T cells engaging anti-CD3/anti-CD28 and ICAM-1 SLBs. Video 2 shows live-cell F-actin dynamics in CAR-expressing Jurkat T cells stimulated via cognate antigen on CD19/CD22 and ICAM-1 SLBs. Video 3 shows live-cell F-actin dynamics in CAR-expressing Jurkat T cells stimulated via TCR using anti-CD3/anti-CD28 and ICAM-1 SLBs.

## Supplementary Material

Review History

## Data Availability

The data underlying Figs. 1, 2, and 3 are available in the published article and its online supplemental information.
