# Supplementary material for: Super-resolution imaging reveals altered actin architecture at CAR-T cell immune synapses
Source: J Cell Biol. 2026 Aug 5;225(10):e202506119. doi: 10.1083/jcb.202506119 (PMC13440243; doi:10.1083/jcb.202506119)
Supplement: Supplementary file 1 [file jcb_202506119_review_history.pdf]

JCB

Super-resolution imaging reveals altered actin architecture at CAR-T cell immune synapses

Olivia Dalby, Efstratios Kirtsios, Hale-Seda Radoykova, Cecilia Zaza, Megan Joseph, Persis Amrolia, Alice Giustacchini, and Sabrina Simoncelli

Corresponding Author(s): Sabrina Simoncelli, University College London

Review Timeline:

Submission Date:	2025-06-18
Editorial Decision:	2025-08-05
Revision Received:	2026-02-25
Editorial Decision:	2026-05-03
Revision Received:	2026-05-11

Monitoring Editor: Ira Mellman

Scientific Editor: Dan Simon

Transaction Report:

(Note: With the exception of the correction of typographical or spelling errors that could be a source of ambiguity, letters and reports are not edited. The original formatting of letters and referee reports may not be reflected in this compilation.)

DOI: https://doi.org/10.1083/jcb.202506119

August 5, 2025

Re: JCB manuscript #202506119

Sabrina Simoncelli
University College London

Dear Dr. Simoncelli,

Thank you for submitting your manuscript entitled "Super-resolution imaging reveals altered actin architecture at CAR-T cell immune synapses." The manuscript has been evaluated by expert reviewers, whose reports are appended below. Unfortunately, after an assessment of the reviewer feedback, our editorial decision is against publication in JCB.

You will see that one reviewer is supportive and only requests minor changes to text and figures. However, the other two reviewers raise substantial concerns about the degree of conceptual advance and the strength of conclusions that can be drawn from the current data.

Although your manuscript is intriguing and timely, we feel that the points raised by the reviewers are more substantial than can be addressed in a typical revision period. If you wish to expedite publication of the current data, it may be best to pursue publication at another journal.

However, given our interest in the topic and in your research, we would be open to resubmission to JCB of a significantly revised manuscript that fully addresses the reviewers' concerns and is subject to further peer-review. Such a revision would need to provide new data addressing Reviewer#1's comments about missing controls and the need for additional imaging studies of the actin-rich protrusions as well as Reviewer#3's concern about small sample sizes for quantitative analysis of actin distribution. A revision would also need to address all other comments including additional discussion of the differences and similarities between the artificial synapse system used here and physiological immune synapses.

Please let us know if you think you would be able to address the major issues outlined above and wish to submit an appeal. We suspect that a substantial amount of additional experimental data may be needed to satisfactorily address the concerns of the reviewers. If you would like to resubmit this work to JCB, please contact the journal office to discuss an appeal of this decision or you may submit an appeal directly through our manuscript submission system. Please note that priority and novelty would be reassessed at resubmission.

Regardless of how you choose to proceed, we hope that the comments below will prove constructive as your work progresses. We would be happy to discuss the reviewer comments further once you've had a chance to consider the points raised in this letter. You can contact the journal office with any questions at cellbio@rockefeller.edu.

Thank you for thinking of JCB as an appropriate place to publish your work.

Sincerely,

Ira Mellman, PhD
Monitoring Editor
Journal of Cell Biology

Dan Simon, PhD
Scientific Editor
Journal of Cell Biology

Reviewer #1 (Comments to the Authors (Required)):

The authors investigate some aspects of F-actin polymerisation in CAR-T cell immunological synapses. They use a supported lipid bilayer platform F-actin using STED microscopy and F-actin spikes and clearances in the IS. They find that CAR-T immunological synapse are slower to evolve and have less clearance of central F-actin and have more F-actin "spikes" at the out margin of the contact area. While this is the first work that I'm aware of that has focused on this issue with STED, other work has called attention to the lower degree of F-actin learning. They should site PMID: 32643825 as they also note the lack of central F-actin clearance, but were not focused on this. They didn't have sufficient resolution to resolve projections/retraction fibers, in the periphery.

Figure 1 characterises the response of cells in the system. There are number of questions I have about this set up. First, the positive control of the study is non-CAR transduced T cell activated by SLB presenting anti-CD3 antibodies. This is a bit confusing as most in the field would not emphasise that the cells lack a CAR, but that they are being functionally stimulated by a substrate attached anti-CD3 antibody. The ERK activation is a good readout for T cell activation and the activation process overall is specific to the CAR. There are two missing controls that are important for all three figures in the study. 1. What do the CAR T cells look like when they are interacting with ICAM-1 only substrates and is this different than the appearance of untransduced T cells. This is not an idle question, but very important as CAR's are known to generate basal signals and thus may respond very differently to ICAM-1 alone in an SLB. 2. Would CAR-T transduction impact the response to anti-CD3 antibodies. The very presence of the CAR may change the way the T cells can respond to anti-CD3. The differences in the IS are attributed to differences in CAR signalling, but they may in fact apply to any activating ligand engaging a CAR-T cell. This should also be investigated with respect to phenotype shown in Figures 2 and 3.
The methods applied in Figure 2 and 3- STED imaging of Jaz staining in fixed cells- is a good way to quantify events at a small number of time points, but the nature of the protrusions and depletion regions will not be clear until dynamics assessments are made. Lifeact is a good way to look at F-actin based protrusion in live cells and be transducer along with the CAR. If one can investigate these dynamically with STED in live cells or using a method like TIRF-SIM to resolve the protrusion then it can be determined if the appearance or protrusion is actually based on protrusion formation or my persistent attachment following contraction of the cell overall (retraction fibers). Similarly, are the F-actin clearing stable and are they associated with exocytosis. Given the current state of the art these questions should be addressed and this could be done through supplemental movies and analysis.

Reviewer #2 (Comments to the Authors (Required)):

This paper addresses the interesting topic of differences in TCR- and CAR-activated immunological synapse architecture and is also one of the few to compare T cells expressing a single, or dual CAR constructs with different specificities. The authors investigate the structure and temporal progression of actin rearrangement at the CAR T cell immunological synapse with the rigor of the best-practice techniques in supported lipid bilayer, imaging and CAR T cell biology. The results demonstrate a difference in the progression from early interactions to late synapse formation between CAR and TCR stimulated T cells. The authors conclude that in CAR-activated T cells, remodelling occurs more abruptly and with more peripheral microvilli. Overall I found this manuscript to be well written, methodologically rigorous, and presenting interesting and well-supported conclusions.

Main points (please address before publication):

- It should be noted that the CARs are 41BB / CD3zeta CARs and the reference is stimulation through the TCR and CD28 costimulatory receptors. Some differences may relate to the difference in co-stimulus given by 41BB and CD28, which should be discussed.

- Fig 2f: This panel was referenced in the text but I could not see it in figure 2.

Minor points (suggested improvements to enhance clarity):

- Although it is correctly asserted that there is no data on whether T cells expressing either one CAR or two CARs have different cytoskeletal remodelling during synapse formation, there is also not a clear reason to believe there would be. Indeed, the data suggested that there were no differences beyond a modest increase in sensitivity (which could be attributed to there effectively being twice the concentration of ligands in the bilayer for the dual CAR-expressing cells). The lack of clear differences could be referred to in a short line in the conclusion, if for no other reason than to justify that the three cell populations were distinguished in most of the presented data.

- Fig 1b: Was the surface expression level of both constructs comparable? Did surface expression of both CARs differ in the single or double positive populations (possibly due to saturated surface trafficking machinery)? The reporter genes don't necessarily correlate with surface expression level, suface expression could be measured with tetramers made by incubating the same biotinylated ligands you used for bilayer experiments with a fluorescent streptavidin, and comparing the binding in FACS. If you have frozen aliquots of transduced cells this would seem worth doing, but given the lack of clear differences between the populations it would not be essential to perform new transductions to test this.

- Fig 1d: Is the ligand density reported here a combination of both antigens? What I mean to say is does the 20 mol/um2 in the figure refer to 20 mol/um2 CD19, and 20 mol/um2 CD22, or 10 mol/um2 of each?

- The affinity of the OKT3 antibody reported (0.5 nM) is on the high side compared with other reports. Kjer-Nielson et al. suggest 2.6 uM, 2004, PNAS (www.pnas.org/cgi/doi/10.1073/pnas.0402295101), Acro biosystems (https://www.acrobiosystems.com/P3220-Human-CD3-epsilon--CD3-delta-Heterodimer-Protein-His-TagTag-Free-%28MALS-verified%29.html) suggest 40 nM with a relatively fast off rate.

- The whiskers on the box-plots throughout the figures are a little excessive when you already have every data point also shown. The whiskers could be removed for enhanced visual clarity.

- T cells initially probe the surface of the APC with microvilli before initial signals from the TCR initiate spreading, adhesion and synapse formation. Observed differences in microvilli are interpreted as accelerated late-stage synapse formation, but considering the delay in actin clearing might it not be described as disorganized and incomplete progression through spreading and contraction phases with retention of initial microvilli structures from the initial contact?

Reviewer #3 (Comments to the Authors (Required)):

The authors of the submitted paper: "Super-resolution imaging reveals altered actin architecture at CAR-T cell immune synapses Actin reorganization at the CAR-T cell synapse" present data from a series of experiments aimed to compare actin organization in the immunological synapse (IS) formed as a result of T cell activation through synthetic chimeric antigen receptors (CARs) and through the T cell receptor (TCR). The authors first present an experiment used to determine the appropriate density of CAR ligands embedded in supported lipid bilayers (SLBs) necessary to activate pERK signaling in T cells. Once the ligand density was determined, the authors compared phenotype of actin in IS generated by T cells on SLBs following ligation of CARs vs. TCRs at three separate time points: 1, 3, and 8 minutes. In this way, the authors can assess dynamics of IS formation and general morphology at different time points. The authors describe a few phenotypic differences observed in their experiment, the main ones being an apparent delay in the maturation of actin phenotype in the CAR T cells, a lack of actin foci in the center of the IS of CAR T cells, differences in cortical density at the IS, and the presence of filopodial projections from the periphery of the IS in CAR T cells.
It is the opinion of this author that the results presented in this paper do not present a significant advancement of the state of knowledge on the subject of actin organization at the IS to justify publishing in JCB. The authors make no attempt at mechanistic insight into the biology described. The actin phenotype in both cells are described and followed with conjecture about how the phenotype could affect CAR-T cell functionality. To this end, the actin phenomena observed by the authors are not novel, and have mostly been described by other authors in previous works (see refs 15, 20, and 27). The experimental reliance on artificial synapse (SLBs) to compare morphology has limited relevance to the physiology of actual IS formation and results from these studies cannot be extended to the functional cytotoxicity of CAR T cells vs. killing resulting from normal TCR stimulation. Further, the paper has a general lack of rigor, and it appears that figures 2 and 3 are prepared from a single set of experiments to image actin morphology.
I do not think any revision or additional experiments will bring this work to a level needed to publish in JCB.

Response to Reviewer Comments

Journal of Cell Biology ID 202506119

Reviewer's comments in italic

Author's responses in blue. Changes to the Manuscript in red.

Reviewer 1

Point 1: *The authors investigate some aspects of F-actin polymerisation in CAR-T cell immunological synapses. They use a supported lipid bilayer platform F-actin using STED microscopy and F-actin spikes and clearances in the IS. They find that CAR-T immunological synapse are slower to evolve and have less clearance of central F-actin and have more F-actin "spikes" at the out margin of the contact area. While this is the first work that I'm aware of that has focused on this issue with STED, other work has called attention to the lower degree of F-actin learning. They should site PMID: 32643825 as they also note the lack of central F-actin clearance, but were not focused on this. They didn't have sufficient resolution to resolve projections/retraction fibers, in the periphery.*

Response 1: We thank the reviewer for bringing these relevant reference to our attention. We have now included the suggested citation (PMID: 32643825) in the introduction section to provide appropriate context to our findings.

The following sentences have been added in the Introduction, page 3: "Consistent with these observations, Dong et al. [17] further reported that CAR-T cells display limited central F-actin clearance. Intriguingly, the same study demonstrated that CAR signaling can proceed independently of linker for activation of T cells (LAT), a scaffold protein essential for TCR-mediated actin reorganization, suggesting that CARs engage a rewired signaling network to coordinate cytoskeletal dynamics and activation."

Point 2: *Figure 1 characterises the response of cells in the system. There are number of questions I have about this set up. First, the positive control of the study is non-CAR transduced T cell activated by SLB presenting anti-CD3 antibodies. This is a bit confusing as most in the field would not emphasise that the cells lack a CAR, but that they are being functionally stimulated by a substrate attached anti-CD3 antibody. The ERK activation is a good readout for T cell activation and the activation process overall is specific to the CAR. There are two missing controls that are important for all three figures in the study. 1. What do the CAR T cells look like when they are interacting with ICAM-1 only substrates and is this different than the appearance of untransduced T cells. This is not an idle question, but very important as CAR's are known to generate basal signals and thus may respond very differently to ICAM-1 alone in an SLB. 2. Would CAR-T transduction impact the response to anti-CD3 antibodies. The very presence of the CAR may change the way the T cells can respond to anti-CD3. The differences in the IS are attributed to differences in CAR signalling, but they may in fact apply to any activating ligand engaging a CAR-T cell. This should also be investigated with respect to phenotype shown in Figures 2 and 3.*

Response 2: We appreciate the reviewer's suggestion to include additional controls. In response, we included data of pERK activation experiments for both CAR-T cells and untransduced T cells interacting with either (i) anti-CD3/anti-CD28 + ICAM-1 SLBs or (ii) ICAM-1 only SLBs.

Our results show no significant difference in pERK activation between CAR-T cells and untransduced T cells under any of these stimulation conditions (Figure R1, new **Figure 1f**). Specifically, CAR expression did not alter basal signalling on ICAM-1-only SLBs, nor did it modify TCR-mediated activation upon anti-CD3/anti-CD28 engagement. These data indicate that CAR expression does not enhance basal signalling activity or interfere with proximal TCR signalling cascades in our experimental system.

Given that pERK activation is a sensitive and early readout of T cell receptor signalling, the absence of any detectable difference strongly suggests that TCR-mediated activation and its downstream processes are not fundamentally affected by CAR expression. Consequently, we would not expect actin remodelling phenotypes or other functional outcomes associated with TCR engagement to differ between CAR-T and untransduced T cells under these control conditions.

For this reason, we did not perform additional STED-based actin phenotyping under ICAM-1–only or anti-CD3 conditions, as the absence of differences in proximal TCR signalling argues against a CAR-dependent alteration of actin remodelling in these contexts. Instead, we focused the fixed-cell STED analyses on conditions directly relevant to CAR engagement and the mechanistic objectives of the study.

Importantly, in the live-cell imaging experiments described in Response 3, we included CAR-T cells stimulated via anti-CD3/anti-CD28 as a functional control. Under TCR-mediated activation, CAR-T cells were indistinguishable from untransduced T cells, including normal reduction of the normalized perimeter-to-area ratio and progression toward synapse maturation. These data further support the conclusion that the altered actin architecture observed in Figures 2 and 3 is specifically driven by antigen-dependent CAR signalling rather than by CAR expression per se or by a generalized alteration in T cell responsiveness.

The new pERK control data have been included in the revised manuscript (see new Figure 1f), and the Results and Discussion sections have been updated accordingly.

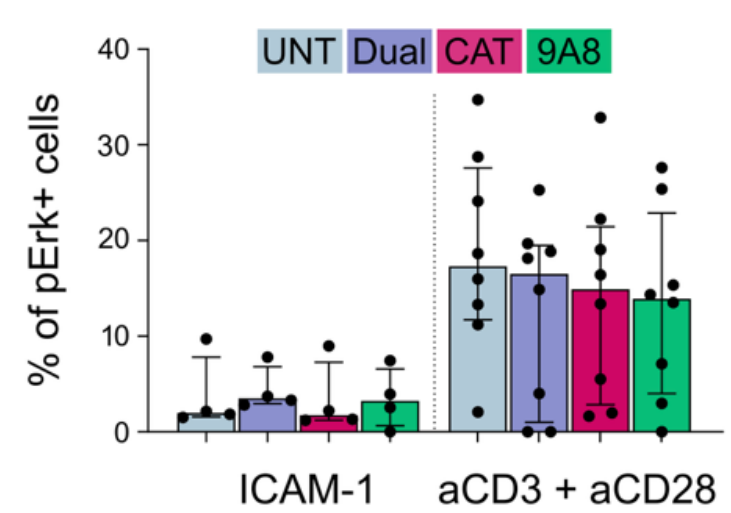

Figure R1 (new Figure 1f). Analysis of confocal pERK data representing % of cells expressing pERK interacting with SLB functionalized with ICAM-1 or anti-CD3 with ICAM-1 on the SLB for untransduced T cells (blue), and dual (purple), CAT (red) and 9A8 (green) CAR-T cells. Each black circle represents one experimental repeat, with bars representing median with interquartile range.

Specifically, the description of Figure 1f in the Results section (page 5, 3rd and 4th paragraph) is as follows:

"To address potential basal signaling in CAR-T cells, we also examined ERK phosphorylation in the absence of specific antigen engagement. Both CAR-T and untransduced T cells were incubated on SLBs functionalized either with ICAM-1 alone or with anti-CD3, anti-CD28 and ICAM-1. Quantification of pERK-positive cells revealed no significant difference between CAR-T and untransduced T cells under any of these two conditions (Figure 1f). These results indicate that CAR expression, whether single or dual, does not enhance basal signaling upon ICAM-1 engagement, nor does it alter the proximal TCR signaling response triggered by anti-CD3/anti-CD28 stimulation. Thus, CAR expression does not appear to interfere with canonical TCR-mediated activation in our experimental system.

Together, these findings confirm that CAR-T cells maintain normal basal and TCR-dependent signaling properties. Consequently, the phenotypic and cytoskeletal differences described in subsequent figures can be attributed to antigen-specific CAR signaling rather than nonspecific effects of CAR expression or altered responsiveness to TCR ligation."

Point 3: *The methods applied in Figure 2 and 3- STED imaging of Jaz staining in fixed cells- is a good way to quantify events at a small number of time points, but the nature of the protrusions and depletion regions will not be clear until dynamics assessments are made. Lifeact is a good way to look at F-actin based protrusion in live cells and be transducer along with the CAR. If one can investigate these dynamically with STED in live cells or using a method like TIRF-SIM to resolve the protrusion then it can be determined if the appearance or protrusion is actually based on protrusion formation or my persistent attachment following contraction of the cell overall (retraction fibers). Similarly, are the F-actin clearing stable and are they associated with exocytosis. Given the current state of the art these questions should be addressed and this could be done through supplemental movies and analysis.*

Response 3: We thank Reviewer 1 for this constructive suggestion. To directly address these points, we performed live-cell imaging of F-actin dynamics using SiR-actin in Jurkat CAR-T and untransduced T cells. Imaging was conducted using spinning-disk confocal microscopy with Optical Photon Reassignment (SoRa), providing enhanced lateral resolution (230 $\pm$ 20 nm, determined by Fourier Ring Correlation), sufficient to resolve peripheral actin structures at the immune synapse. These data are presented in new **Supplementary Figure S3** (see also Figure R2) and **Videos 1–3**.

Live-cell imaging demonstrates that the peripheral actin structures observed in fixed STED images correspond to persistent features rather than transient retraction fibers. Quantitative analysis of normalized perimeter-to-area ratios shows that untransduced (TCR-stimulated) T cells exhibit a progressive decrease over time, consistent with microvilli retraction and normal synapse maturation. In contrast, CAR-T cells stimulated via cognate antigen maintain elevated perimeter-to-area ratios throughout the imaging window, indicating sustained peripheral actin protrusions and incomplete contraction.

To directly link the live-cell and fixed-cell datasets, we expanded **Figure 2f** to include the 1-minute activation time point in the STED analysis (see also Figure R3). At this early time point, both untransduced and CAR-T cells display similarly elevated perimeter-to-area ratios, consistent with initial microvillar probing during early antigen contact. Over time, the ratio decreases in untransduced T cells, reflecting proper contraction and synapse maturation, whereas it remains elevated in CAR-T cells, consistent with persistent microvillar-like structures and incomplete cytoskeletal reorganization.

To determine whether this phenotype reflects antigen-specific CAR signaling rather than an intrinsic consequence of CAR expression, we performed live imaging of CAR-T cells stimulated via anti-CD3/anti-CD28 (TCR-mediated activation). Under these conditions, CAR-T cells behaved indistinguishably from untransduced T cells, including normal reduction of the perimeter-to-area ratio and formation of a stable synapse (**Supplementary Figure S3, Video 3**). These findings demonstrate that the persistent peripheral actin phenotype is specifically driven by cognate antigen engagement of the CAR.

Together, the revised fixed-cell analysis (Figure 2f, now including the 1-minute time point) and the new live-cell imaging data (Supplementary Figure S3, Videos 1-3) support a model in which CAR-T cells activated via cognate antigen engagement fail to complete the normal spreading–contraction cycle of synapse formation, resulting in retention of early microvillar-like actin structures. The Results, Discussion, and Abstract have been revised accordingly.

We believe these additions, now discussed in the Results section, page 7-8, directly address the reviewer's concerns regarding actin dynamics and substantially strengthen the mechanistic integration of the fixed super-resolution and live-cell datasets.

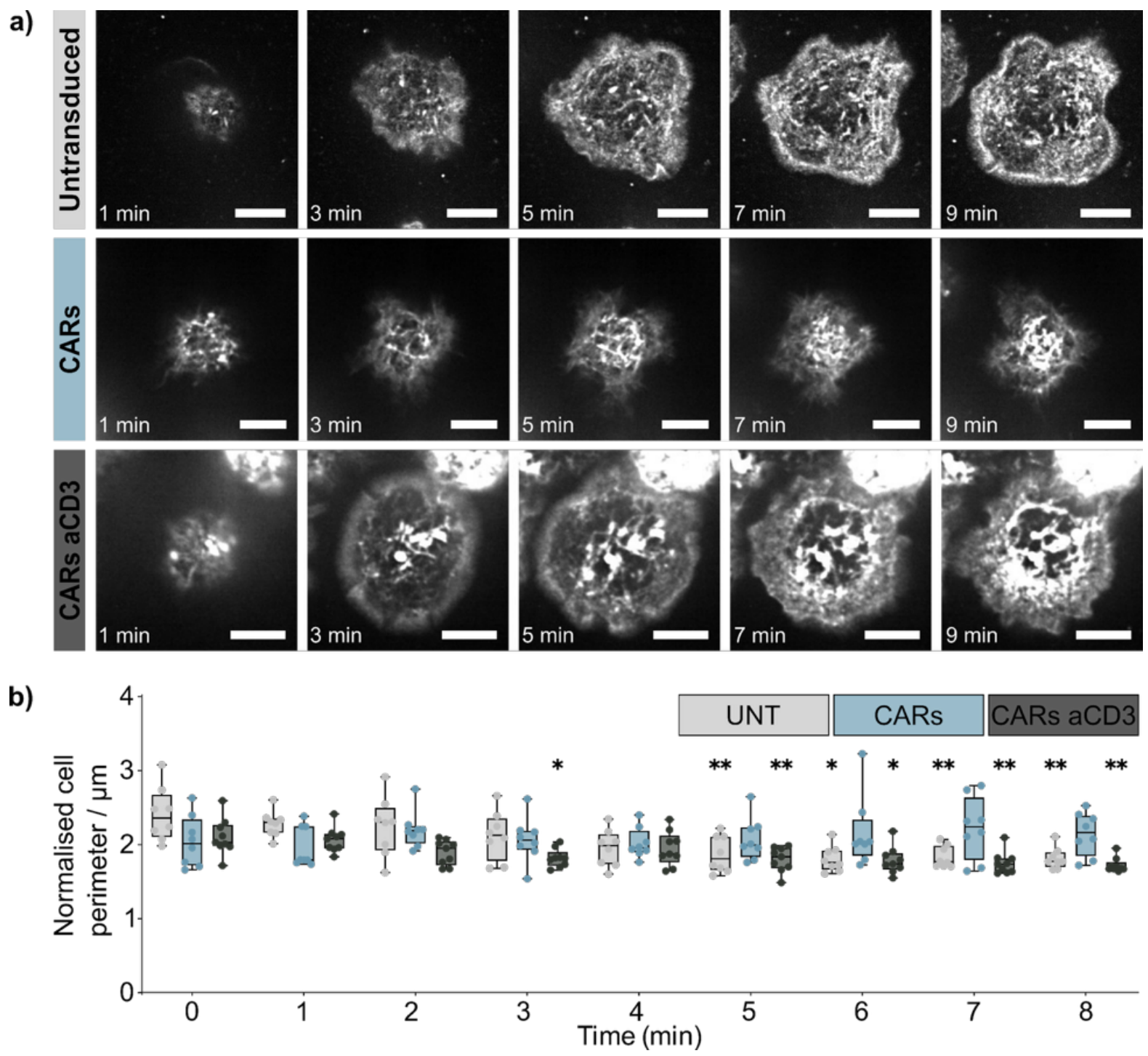

Figure R2 (new Supplementary Figure S3). (a) Representative still frames from live-cell imaging of untransduced Jurkat T cells (blue, top) and CAR-expressing Jurkat T cells (grey, middle and bottom) labeled with SiR-actin and interacting with SLB. SLBs were functionalized with anti-CD3/anti-CD28 and ICAM-1 to stimulate untransduced T cells (top) or CAR-T cells via the endogenous TCR (bottom), or with CD19/CD22 ligands and ICAM-1 to stimulate CAR-T cells via cognate antigen engagement (middle). Images correspond to the basal synapse plane at specific time points following initial contact. Scale bars, 5 μm. (b) Quantification of normalized cell perimeter over time for untransduced T cells stimulated with anti-CD3/anti-CD28 and ICAM-1 SLBs (blue), CAR-T cells stimulated via cognate antigen engagement on CD19/CD22- and ICAM-1 functionalized SLBs (grey), and CAR-T cells stimulated via TCR activation on anti-CD3/anti-CD28 and ICAM-1 SLBs (dark grey). The perimeter-to-area ratio reports the temporal dynamics of peripheral actin protrusions during immunological synapse formation. $n > 8$ cells per condition pooled from 3 independent experiments. Corresponding full time-lapse sequences are shown in Videos 1–3.

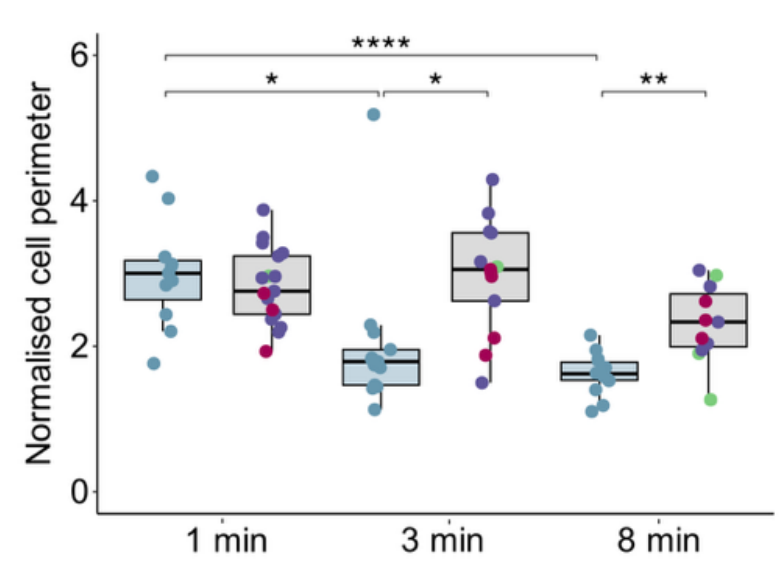

Figure R3 (updated Figure 2f). Quantitative analysis of STED actin images representing 1-, 3- and 8-minute cell synapses showing the normalized cell perimeter for both untransduced (blue) and CAR-T (grey) cells. $n > 12$ CAR-T or untransduced T cells for each activation condition.

Reviewer: 2

This paper addresses the interesting topic of differences in TCR- and CAR-activated immunological synapse architecture and is also one of the few to compare T cells expressing a single, or dual CAR constructs with different specificities. The authors investigate the structure and temporal progression of actin rearrangement at the CAR T cell immunological synapse with the rigor of the best-practice techniques in supported lipid bilayer, imaging and CAR T cell biology. The results demonstrate a difference in the progression from early interactions to late synapse formation between CAR and TCR stimulated T cells. The authors conclude that in CAR-activated T cells, remodelling occurs more abruptly and with more peripheral microvilli. Overall I found this manuscript to be well written, methodologically rigorous, and presenting interesting and well-supported conclusions.

Response: We sincerely thank Reviewer 2 for their thoughtful and positive evaluation of our manuscript. We are grateful for the recognition of our study's focus on comparing TCR- and CAR-activated immunological synapse architecture, including the rare examination of single versus dual CAR constructs with different specificities. We appreciate the acknowledgement of our methodological rigor in employing supported lipid bilayers, advanced imaging, and established CAR T cell biology techniques. We also appreciate the constructive criticisms, which have guided us in further strengthening the manuscript through additional experimentation and clarification.

Main points (please address before publication):

Point 1*: It should be noted that the CARs are 41BB / CD3zeta CARs and the reference is stimulation through the TCR and CD28 costimulatory receptors. Some differences may relate to the difference in co-stimulus given by 41BB and CD28, which should be discussed.*

Response 1: We thank the reviewer for this thoughtful comment. We have now expanded the Results section ("CAR-T cells exhibit abrupt cytoskeletal remodeling...") to explicitly address the potential impact of differences in co-stimulatory signaling between CAR- and TCR-mediated activation. Specifically, we discuss that while 4-1BB and CD28 are known to engage distinct intracellular pathways and metabolic programs, comparative studies of anti-HER2 and κ-CARs containing either CD28ζ or 4-1BBζ signaling domains have shown, via diffraction limited experiments, similarly altered actin architectures. Thus, we now highlight that although costimulatory differences may modulate the kinetics of cytoskeletal remodeling, the overall actin phenotype observed here, delayed clearance and denser central actin, likely reflects a shared feature of CAR-mediated signaling rather than a specific effect of the costimulatory domain.

The following text was added to the Results section (page 7, 2nd paragraph):

"These findings suggest that whilst CAR-T cells ultimately form actin structures resembling classical synapses, they do so with delayed kinetics and retain a denser central actin mesh. We attribute these differences primarily to CAR-receptor geometry and antigen-binding context. However, we note that our CARs employ a 4-1BB (CD137) costimulatory module, whereas the untransduced T-cell reference uses CD28. Although 4-1BB and CD28 signaling differ in pathway utilization and metabolic programming,[21,22] both anti-HER2 CARs with CD28ζ and

Kappa-CARs with either CD28ζ or 4-1BBζ have been reported to exhibit similarly altered actin architectures.[15,16] This suggests that the distinct cytoskeletal remodeling observed here likely reflects a shared property of CAR-mediated signaling rather than a specific effect of the costimulatory domain."

Point 2*: Fig 2f: This panel was referenced in the text but I could not see it in figure 2.*

Response 2: We thank the reviewer for noting this oversight. The label "f" was inadvertently missing from Figure 2 in the submitted version.

We have now added the correct label to ensure the panel is clearly identifiable in the revised figure.

Minor points (suggested improvements to enhance clarity):

Point 3*: Although it is correctly asserted that there is no data on whether T cells expressing either one CAR or two CARs have different cytoskeletal remodelling during synapse formation, there is also not a clear reason to believe there would be. Indeed, the data suggested that there were no differences beyond a modest increase in sensitivity (which could be attributed to there effectively being twice the concentration of ligands in the bilayer for the dual CAR-expressing cells). The lack of clear differences could be referred to in a short line in the conclusion, if for no other reason than to justify that the three cell populations were distinguished in most of the presented data.*

Response 3: We thank the reviewer for this helpful suggestion. We have now added a brief statement in the concluding section to clarify that, while single- and dual-CAR-T cells were analyzed separately throughout for completeness, their actin-remodeling profiles were highly similar under high-antigen conditions. The added text (page 10, last paragraph) reads:

"We note that, although we distinguished single- and dual-CAR-expressing cells throughout our analyses, their cytoskeletal remodeling dynamics and synapse morphologies were largely comparable. We therefore conclude that the principal cytoskeletal features described here are generalizable across single- and dual-CAR-T populations."

This addition acknowledges the reviewer's point and clarifies our rationale for distinguishing the three cell populations in the presented analyses.

Point 4*: Fig 1b: Was the surface expression level of both constructs comparable? Did surface expression of both CARs differ in the single or double positive populations (possibly due to saturated surface trafficking machinery)? The reporter genes don't necessarily correlate with surface expression level, suface expression could be measured with tetramers made by incubating the same biotinylated ligands you used for bilayer experiments with a fluorescent streptavidin, and comparing the binding in FACS. If you have frozen aliquots of transduced cells this would seem worth doing, but given the lack of clear differences between the populations it would not be essential to perform new transductions to test this.*

Response 4: We thank the reviewer for this insightful comment regarding potential differences in surface expression between the two CAR constructs, particularly within single- versus dual-transduced populations.

Both CAR constructs are encoded in bicistronic vectors in which the CAR and its corresponding fluorescent reporter are separated by a T2A self-cleaving peptide under a single promoter. This design results in equimolar translation of CAR and reporter at the transcript level, providing a mechanistic expectation that reporter fluorescence should scale proportionally with CAR production. We recognize, however, that post-translational processes—including folding efficiency, ER trafficking, and membrane stability—could in principle uncouple reporter intensity from surface CAR density. Therefore, direct measurement of surface CAR was important to validate reporter-based inference.

In the original dataset (Figure 1b), surface CAR was inferred from fluorescent reporter intensity. For the CAT construct, we compared reporter fluorescence between CAT single-positive cells (mCherry^{+} GFP^{-}) and CAT-positive cells within the dual-transduced population (mCherry^{+} GFP^{+}). Analogously, for the 9A8 construct, we compared reporter fluorescence between 9A8 single-positive cells (GFP^{+} mCherry^{-}) and 9A8-positive cells within the dual-transduced population (GFP^{+} mCherry^{+}). Reporter intensity distributions were similar between single- and dual-positive populations, suggesting comparable expression.

We agree that direct tetramer-based quantification of surface CAR levels would provide an orthogonal validation. However, we did not have sufficient frozen aliquots from the original donor samples used in this work to perform additional tetramer staining on the same biological material (i.e., we prioritize the available stocks to answer Reviewer's 1, Point 2). To address this, we performed instead a validation experiment in an independent donor sample. Here, dual- and single-transduced T cells were analyzed using anti-idiotypic antibodies recognizing the extracellular scFv domains of CAT-CD19 and 9A8-CD22, directly quantifying surface CAR levels. This analysis is now included in Figure R4. For the CAT construct, we compared geometric mean fluorescence intensity (MFI) between CAT single-positive cells (mCherry^{+} GFP^{-}) and CAT-positive cells within the dual-transduced population (mCherry^{+} GFP^{+}). Analogously, for the 9A8 construct, we compared 9A8 single-positive cells (GFP^{+} mCherry^{-}) and 9A8-positive cells within the dual-transduced population (GFP^{+} mCherry^{+}). As shown in Figure R4c, surface CAR MFI was comparable between single- and dual-positive populations for both constructs, indicating that co-expression of a second CAR does not measurably reduce surface levels of the first construct within the analyzed range.

To directly test whether reporter intensity accurately reflects surface CAR abundance, we further performed binning analysis in the independent donor sample. Double-positive cells were stratified into logarithmic bins based on reporter fluorescence intensity, and surface CAR MFI was quantified within each bin. For both constructs, surface CAR staining scaled proportionally with reporter intensity across the dynamic range (Figure R4d–e). This confirms that, in our system, reporter fluorescence serves as a reliable proportional readout of relative surface CAR expression.

We fully acknowledge that fluorescent reporter intensity cannot be used to infer absolute CAR molecule numbers. However, the independent validation demonstrates concordant scaling between reporter and surface staining, supporting the use of reporter intensity to compare

relative CAR expression levels between single- and dual-positive populations. Importantly, these data confirm that expression of one CAR does not substantially alter the surface abundance of the second construct. Together, these analyses address the reviewer's concern and support the conclusion that surface expression levels of each CAR are comparable between single- and dual-transduced cells in our experimental system.

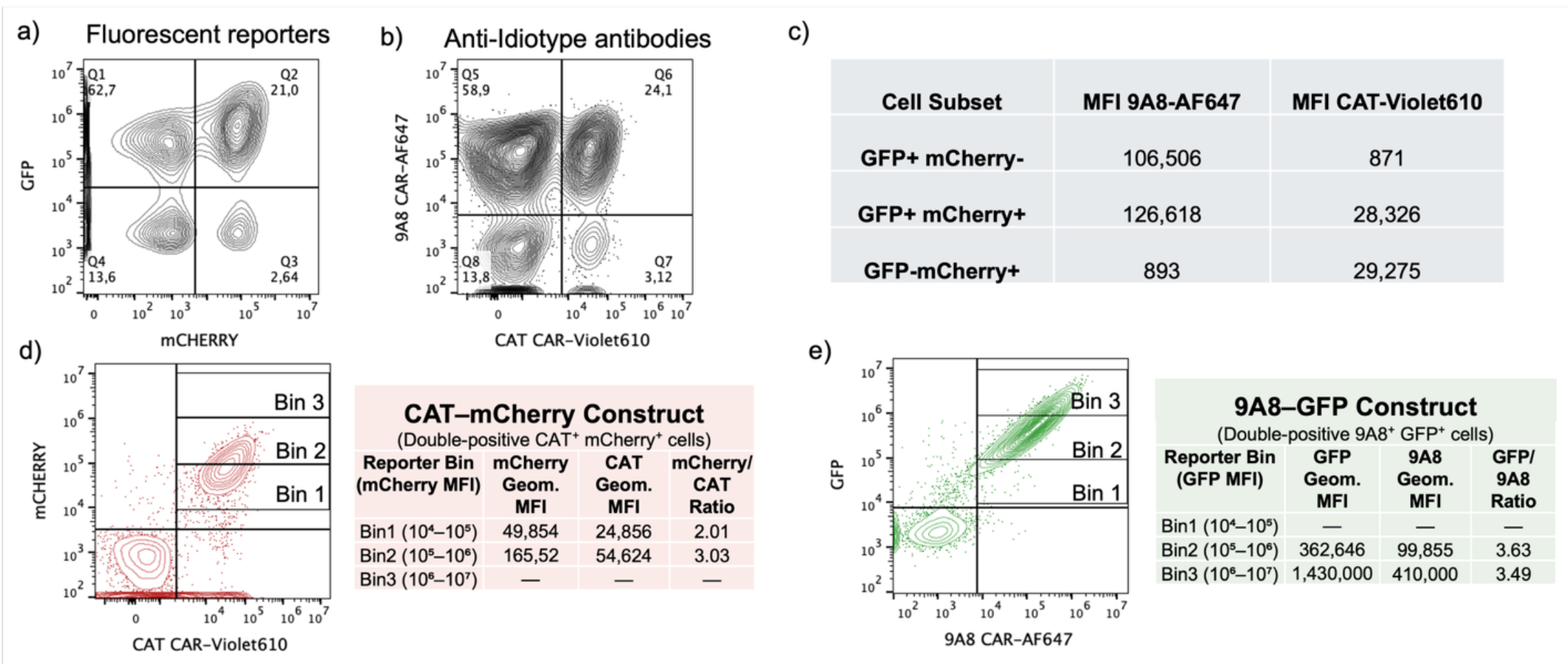

Figure R4. Correlation between fluorescent reporter intensity and surface CAR expression**. (a-b)** Representative flow cytometry plots showing that the use of fluorescent reporters (GFP, mCherry) yields quantification of CAR T cell subsets nearly identical to that obtained via direct antibody staining (anti-9A8, anti-CAT anti-idiotype antibodies), with overlapping signals confirming that the reporter accurately identifies the CAR-expressing population. (**c**) Geometric mean fluorescence intensity (MFI) of surface CAR staining (anti-idiotype antibody with fluorescent secondary detection) was quantified in single- and dual-transduced populations. For CAT, MFI was compared between CAT single-positive cells (mCherry$^+$ GFP$^-$) and CAT-positive cells within the dual-transduced population (mCherry$^+$ GFP$^+$). For 9A8, MFI was compared between 9A8 single-positive cells (GFP$^+$ mCherry$^-$) and 9A8-positive cells within the dual-transduced population (GFP$^+$ mCherry$^+$). Surface CAR levels were comparable between single- and dual-positive cells for both constructs. **(d-e)** Representative flow cytometry plots showing the co-expression of GFP/9A8 CAR and mCherry/CAT CAR. Cells were gated into logarithmic bins based on reporter MFI to assess the relationship between intracellular reporter levels and surface CAR display.

Point 5*: Fig 1d: Is the ligand density reported here a combination of both antigens? What I mean to say is does the 20 mol/um2 in the figure refer to 20 mol/um2 CD19, and 20 mol/um2 CD22, or 10 mol/um2 of each?*

Response 5: We thank the reviewer for seeking clarification on this point. The ligand density reported in Fig. 1d refers to 20 molecules/μm² for each antigen individually, meaning 20 molecules/μm² CD19 and 20 molecules/μm² CD22 when both ligands are present.

We have clarified this explicitly in the figure legend to avoid ambiguity.

Point 6*: The affinity of the OKT3 antibody reported (0.5 nM) is on the high side compared with other reports. Kjer-Nielson et al. suggest 2.6 uM, 2004, PNAS (www.pnas.org/cgi/doi/10.1073/pnas.0402295101), Acro biosystems*

(https://www.acrobiosystems.com/P3220-Human-CD3-epsilon--CD3-delta-Heterodimer-Protein-His-TagTag-Free-%28MALS-verified%29.html) suggest 40 nM with a relatively fast off rate.

Response 6: We thank the reviewer for pointing out the discrepancy in the reported affinity of the anti-CD3 antibody (OKT3). We agree that our previously cited value (0.5 nM) was on the high side compared with other published measurements.

We have therefore revised the manuscript to reflect the suggested literature value of 2.6 μM and have cited the corresponding reference. This correction does not alter any of our interpretations or conclusions.

Point 7*: The whiskers on the box-plots throughout the figures are a little excessive when you already have every data point also shown. The whiskers could be removed for enhanced visual clarity.*

Response 7: We thank the reviewer for this helpful suggestion regarding figure presentation. We agree that the individual data points provide a direct view of data distribution; however, we have chosen to retain the whiskers in the box-plots because they convey important statistical context. Specifically, the whiskers represent the interquartile range and highlight the spread and variability of the dataset beyond the median, allowing readers to quickly interpret the extent and symmetry of the distribution without relying solely on visual inspection of individual points. This dual representation—individual points and summary statistics—ensures that the figures remain accessible to readers who may be accustomed to interpreting box-plots in a more traditional format, and provides a concise summary for comparing across conditions.

Point 8*: T cells initially probe the surface of the APC with microvilli before initial signals from the TCR initiate spreading, adhesion and synapse formation. Observed differences in microvilli are interpreted as accelerated late-stage synapse formation, but considering the delay in actin clearing might it not be described as disorganized and incomplete progression through spreading and contraction phases with retention of initial microvilli structures from the initial contact?*

Response 8: We thank the reviewer for this insightful comment. We agree with the reviewer interpretation that our data might be more consistent with a model of disorganized or incomplete cytoskeletal contraction rather than accelerated maturation. To address this, we have expanded Figure 2f to include the normalized perimeter at the 1-minute activation time point, showing that both untransduced and CAR-T cells initially display similar perimeter/area ratios, consistent with early microvilli probing. Over time, the normalized perimeter value decreases in untransduced T cells, reflecting proper contraction and synapse maturation, whereas it remains elevated in CAR-T cells, consistent with persistent microvilli and incomplete cytoskeletal reorganization. We also performed new live imaging experiments (new Supplementary Figure 3, Videos 1-3), which are in line with these observations, as described in Response 3 from Reviewer 1.

Specifically, we have included the following text to address the temporal changes of normalized cell perimeter values to the Results section (page 7-8):

"Quantification of normalized perimeter (Figure 2f) revealed that both CAR-T and untransduced T cells exhibited similarly high values at 1 minute, consistent with the presence of initial

microvilli probing during early antigen contact. In untransduced T cells, the ratio decreased over time (p-values ≤ 0.01, for 1 minute compared to both 3 and 8 minutes), reflecting normal contraction and retraction of microvilli as the synapse matured. In contrast, CAR-T cells retained elevated normalized perimeter at 3 and 8 minutes which were significantly higher than untransduced cells (p-values = 0.03 and 0.01, respectively; Figure 2f), indicating persistent peripheral protrusions. These data suggest that CAR-T cells fail to fully contract and reorganize the actin network, leading to retention of initial microvillar-like structures.

To complement these static snapshots, we performed live-cell imaging of Jurkat CAR-T and untransduced Jurkat T cells labelled with SiR-actin to visualize F-actin dynamics during engagement with activating supported lipid bilayers (Supplementary Figure S3, Video 1–3). Imaging was performed on a Spinning Disk Super-Resolution microscope with Optical Photon Reassignment (SoRa), achieving a lateral resolution of 230 ± 20 nm as determined by Fourier Ring Correlation. Time-resolved analysis confirmed that untransduced T cells exhibited a gradual decrease in cell perimeter-to-area ratio, with significant reductions beginning at 5 min (p-values ≤ 0.011 thereafter, all compared to 1 min), consistent with microvilli retraction and normal synapse maturation (Supplementary Figure S3, Video 1). In contrast, CAR-T cells did not exhibit a significant reduction in perimeter-to-area ratio at any time point within the imaging window (all p-values > 0.19 compared to 1 min), indicating sustained peripheral actin protrusions and failure to undergo normal synapse contraction (Supplementary Figure S3, Video 2). Importantly, to determine whether this phenotype reflects antigen-specific CAR signaling rather than an intrinsic property of CAR expression, we repeated the live-cell imaging experiments of Jurkat CAR-T cells using anti-CD3/anti-CD28 stimulation. Under TCR-mediated activation, CAR-T cells exhibited normal contraction dynamics, with significant reductions in perimeter-to-area ratio beginning at 3 min (p = 0.012; p ≤ 0.022 after 5 min, all compared to 1 min), and formation of a stable synapse (Supplementary Figure S3, Video 3). Thus, the persistent microvillar projections and aberrant cytoskeletal remodeling observed upon engagement of the CAR by its cognate antigen are specifically driven by antigen-dependent CAR signaling and are not elicited by canonical TCR activation in CAR-expressing cells.

Together, the live-cell and fixed-cell data indicate that CAR-T cells activated through cognate antigen engagement retain initial microvillar structures and fail to complete the normal spreading–contraction cycle, resulting in a disorganized or partially matured immunological synapse architecture.”

Corresponding minor revisions have also been made throughout the Results section, as well as in the Abstract and final summary paragraphs, to accurately reflect this updated interpretation.

Reviewer: 3

The authors of the submitted paper: "Super-resolution imaging reveals altered actin architecture at CAR-T cell immune synapses Actin reorganization at the CAR-T cell synapse" present data from a series of experiments aimed to compare actin organization in the immunological synapse (IS) formed as a result of T cell activation through synthetic chimeric antigen receptors (CARs) and through the T cell receptor (TCR). The authors first present an experiment used to determine the appropriate density of CAR ligands embedded in supported lipid bilayers (SLBs) necessary to activate pERK signaling in T cells. Once the ligand density was determined, the

authors compared phenotype of actin in IS generated by T cells on SLBs following ligation of CARs vs. TCRs at three separate time points: 1, 3, and 8 minutes. In this way, the authors can assess dynamics of IS formation and general morphology at different time points. The authors describe a few phenotypic differences observed in their experiment, the main ones being an apparent delay in the maturation of actin phenotype in the CAR T cells, a lack of actin foci in the center of the IS of CAR T cells, differences in cortical density at the IS, and the presence of filopodial projections from the periphery of the IS in CAR T cells.
It is the opinion of this author that the results presented in this paper do not present a significant advancement of the state of knowledge on the subject of actin organization at the IS to justify publishing in JCB. The authors make no attempt at mechanistic insight into the biology described. The actin phenotype in both cells are described and followed with conjecture about how the phenotype could affect CAR-T cell functionality. To this end, the actin phenomena observed by the authors are not novel, and have mostly been described by other authors in previous works (see refs 15, 20, and 27). The experimental reliance on artificial synapse (SLBs) to compare morphology has limited relevance to the physiology of actual IS formation and results from these studies cannot be extended to the functional cytotoxicity of CAR T cells vs. killing resulting from normal TCR stimulation. Further, the paper has a general lack of rigor, and it appears that figures 2 and 3 are prepared from a single set of experiments to image actin morphology.

I do not think any revision or additional experiments will bring this work to a level needed to publish in JCB.

Response: We thank the reviewer for their detailed feedback and for taking the time to evaluate our manuscript. We appreciate the concerns raised regarding novelty, physiological relevance, and rigor. Below, we respond to each point:

Novelty and advancement of the field: We respectfully disagree with the assessment that the work does not advance current knowledge. While previous studies (refs. 15, 16, 17) have reported aspects of actin organization at CAR-mediated synapses, these analyses were largely performed at diffraction-limited resolution, at later time points, or without a direct, temporally matched comparison to TCR-driven synapses under identical ligand-density conditions.

In the present study, we systematically and quantitatively compare CAR- and TCR-mediated synapses at defined early time points (1, 3, and 8 minutes) using super-resolution imaging. We analyze multiple architectural parameters, including central actin clearance, actin foci distribution, peripheral protrusion number, and cortical mesh density, within the same experimental framework. We do not claim the discovery of entirely new actin structures. Rather, the advance lies in the controlled, side-by-side and time-resolved analysis demonstrating that CAR engagement alters the kinetics and completion of actin remodeling relative to canonical TCR activation.

Importantly, our revised manuscript incorporates live-cell actin imaging (Supplementary Figure S3; Videos 1–3), which directly addresses the dynamic nature of these phenotypes. These experiments demonstrate that CAR-T cells stimulated via cognate antigen retain elevated perimeter-to-area ratios over time, consistent with persistent peripheral microvillar-like protrusions and incomplete contraction. In contrast, CAR-expressing cells stimulated via anti-CD3/anti-CD28 are indistinguishable from untransduced T cells and undergo normal spreading

followed by contraction. Thus, the altered architecture observed in fixed STED images reflects antigen-specific CAR signaling and corresponds to a delayed or incomplete progression through the canonical spreading–contraction cycle, rather than a fundamentally distinct activation state or an intrinsic defect in CAR-expressing cells.

To our knowledge, this integrated super-resolution and live-cell comparison defining altered remodeling kinetics at early stages of synapse formation has not previously been systematically described.

Physiological relevance of SLBs: We acknowledge that supported lipid bilayers are a reductionist system; however, they remain a widely accepted and powerful platform to dissect early events of immune synapse formation with precise ligand control. SLBs have been extensively used in high-impact studies on TCR signaling and cytoskeletal remodeling (i.e., *Science* 1999, doi:10.1126/science.285.5425.221; *Cell* 2017, doi:10.1016/j.cell.2017.03.006; *Nature Comm.* 2023, doi:10.1038/s41467-023-36855-9; *Nature Immunology*. 2020, https://doi.org/10.1038/s41590-020-0719-0). By using identical SLB conditions for both CAR- and TCR-mediated activation, we can isolate and compare intrinsic cytoskeletal differences without the confounding variability inherent in target cell assays.

Rigor and reproducibility: Our imaging dataset is derived from n = 3 independent human donors, where we have analyzed >12 cells for each cell type (CAR-T and untransduced T cells) and each time point (1, 3, and 8 min). This results in a dataset size comparable to or exceeding other high-resolution STED actin imaging studies on Jurkat T cells, such as Yi *et al.* (*Sci. Adv.*, 2016; doi:10.1126/sciadv.1603032), which used a total of 32 cells for STED imaging. Furthermore, we note that each STED acquisition for a single cell required approximately 30 minutes. Given the complexity of the setup, the number of cells imaged represents a substantial and rigorous dataset. Figures 2 and 3 were not generated from a single experiment, but rather from multiple independent experimental repeats across different days and donors. Additionally, we have now included live-cell super-resolution imaging actin dynamics experiments (Supplementary Figure 3, Videos S1-S3), which strengthen reproducibility.

Scope and contribution: We fully recognize that this study is architectural rather than mechanistic. However, defining structural phenotypes at super-resolution with quantitative temporal analysis represents an important step toward understanding how synthetic receptor geometry and signaling context influence cytoskeletal organization. We have revised the manuscript to temper interpretive language and avoid overstating functional implications.

In summary, we believe our study provides a robust, quantitative, and high-resolution comparison of actin remodeling at CAR- and TCR-mediated immune synapses. While mechanistic dissection will require future work, we believe that defining these reproducible structural differences constitutes a meaningful contribution to the field.

May 3, 2026

RE: JCB Manuscript #202506119R-A

Sabrina Simoncelli
University College London

Dear Dr. Simoncelli,

Thank you for submitting your revised manuscript entitled "Super-resolution imaging reveals altered actin architecture at CAR-T cell immune synapses." The manuscript was re-reviewed by two of the original reviewers. We would be happy to publish your paper in JCB pending final revisions necessary to meet our formatting guidelines (see details below). While we share Reviewer 3's interest in understanding the molecular mechanisms better, this is not required for a Report.

To avoid unnecessary delays in the acceptance and publication of your paper, please read the following information carefully.

A. MANUSCRIPT ORGANIZATION AND FORMATTING:

Full guidelines are available on our Instructions for Authors page, https://jcb.rupress.org/submission-guidelines#revised.
Submission of a paper that does not conform to JCB guidelines will delay the acceptance of your manuscript.

1) Text limits: Character count for Reports is < 20,000, not including spaces. Count includes title page, abstract, introduction, results & discussion, and acknowledgments. Count does not include materials and methods, figure legends, references, tables, or supplemental legends. Reports must have a combined 'Results and Discussion' section.

2) Figure formatting: Reports may have up to 5 main text figures. Scale bars must be present on all microscopy images, including inset magnifications. Molecular weight or nucleic acid size markers must be included on all gel electrophoresis. Also, please avoid pairing red and green for images and graphs to ensure legibility for color-blind readers. If red and green are paired for images, please ensure that the particular red and green hues used in micrographs are distinctive with any of the colorblind types. If not, please modify colors accordingly or provide separate images of the individual channels.

3) Statistical analysis: Error bars on graphic representations of numerical data must be clearly described in the figure legend. The number of independent data points (n) represented in a graph must be indicated in the legend. Please indicate whether 'n' refers to technical or biological replicates (i.e. number of analyzed cells, samples or animals, number of independent experiments). If independent experiments with multiple biological replicates have been performed, we recommend using distribution-reproducibility SuperPlots (please see Lord et al., JCB 2020) to better display the distribution of the entire dataset, and report statistics (such as means, error bars, and P values) that address the reproducibility of the findings.

Statistical methods should be explained in full in the materials and methods. For figures presenting pooled data the statistical measure should be defined in the figure legends. Please also be sure to indicate the statistical tests used in each of your experiments (both in the figure legend itself and in a separate methods section) as well as the parameters of the test (for example, if you ran a t-test, please indicate if it was one- or two-sided, etc.). Also, if you used parametric tests, please indicate if the data distribution was tested for normality (and if so, how). If not, you must state something to the effect that "Data distribution was assumed to be normal but this was not formally tested."

4) Materials and methods: Should be comprehensive and not simply reference a previous publication for details on how an experiment was performed. Please provide full descriptions (at least in brief) in the text for readers who may not have access to referenced manuscripts. The text should not refer to methods "...as previously described."

5) For all cell lines, vectors, strains, constructs/cDNAs, etc. - all genetic material: please include database / vendor ID (e.g. Addgene, ATCC, etc.) or if unavailable, please briefly describe their basic genetic features, even if described in other published work or gifted to you by other investigators (and provide references where appropriate). Please be sure to provide the sequences for all of your oligos: primers, si/shRNA, RNAi, gRNAs, etc. in the materials and methods. You must also indicate in the methods the source, species, and catalog numbers/vendor identifiers (where appropriate) for all of your antibodies, including secondary. If antibodies are not commercial, please add a reference citation if possible.

6) Microscope image acquisition: The following information must be provided about the acquisition and processing of images:
a. Make and model of microscope
b. Type, magnification, and numerical aperture of the objective lenses
c. Temperature
d. Imaging medium
e. Fluorochromes

f. Camera make and model
g. Acquisition software
h. Any software used for image processing subsequent to data acquisition. Please include details and types of operations involved (e.g., type of deconvolution, 3D reconstitutions, surface or volume rendering, gamma adjustments, etc.).

7) References: There is no limit to the number of references cited in a manuscript. References should be cited parenthetically in the text by author and year of publication. Abbreviate the names of journals according to PubMed.

8) Supplemental materials: Reports are typically allowed up to 3 supplemental figures and 10 videos. You currently exceed this limit but, in this case, we will be able to give you the extra space. Please also note that tables, like figures, should be provided as individual, editable files. A summary of all supplemental material should appear at the end of the Materials and methods section. Please include one brief sentence per item.

9) Video legends: Should describe what is being shown, the cell type or tissue being viewed (including relevant cell treatments, concentration and duration, or transfection), the imaging method (e.g., time-lapse epifluorescence microscopy), what each color represents, how often frames were collected, the frames/second display rate, and the number of any figure that has related video stills or images.

10) eTOC summary: A ~40-50 word summary that describes the context and significance of the findings for a general readership should be included on the title page. The statement should be written in the present tense and refer to the work in the third person. It should begin with "First author name(s) et al..." to match our preferred style.

11) Conflict of interest statement: JCB requires inclusion of a statement in the acknowledgements regarding competing financial interests. If no competing financial interests exist, please include the following statement: "The authors declare no competing financial interests." If competing interests are declared, please follow your statement of these competing interests with the following statement: "The authors declare no further competing financial interests."

12) A separate author contribution section is required following the Acknowledgments in all research manuscripts. All authors should be mentioned and designated by their first and middle initials and full surnames. We encourage use of the CRediT nomenclature (https://casrai.org/credit/).

13) ORCID IDs: ORCID IDs are unique identifiers allowing researchers to create a record of their various scholarly contributions in a single place. Please note that ORCID IDs are required for all authors. At resubmission of your final files, please be sure to provide your ORCID ID and those of all co-authors.

14) Journal of Cell Biology now requires a data availability statement for all research article submissions. These statements will be published in the article directly above the Acknowledgments. The statement should address all data underlying the research presented in the manuscript. Please visit the JCB instructions for authors for guidelines and examples of statements at (https://rupress.org/jcb/pages/editorial-policies#data-availability-statement).

B. FINAL FILES:

Please upload the following materials to our online submission system. These items are required prior to acceptance. If you have any questions, contact JCB's Managing Editor, Lindsey Hollander (lhollander@rockefeller.edu).

-- An editable version of the final text (.DOC or .DOCX) is needed for copyediting (no PDFs).

-- High-resolution figure and MP4 video files: See our detailed guidelines for preparing your production-ready images, https://jcb.rupress.org/fig-vid-guidelines.

-- Cover images: If you have any striking images related to this story, we would be happy to consider them for inclusion on the journal cover. Submitted images may also be chosen for highlighting on the journal table of contents or JCB homepage carousel. Images should be uploaded as TIFF or EPS files and must be at least 300 dpi resolution.

It is JCB policy that if requested, original data images must be made available to the editors. Failure to provide original images upon request will result in unavoidable delays in publication. Please ensure that you have access to all original data images prior to final submission.

The license to publish form must be signed before your manuscript can be sent to production. A link to the license to publish form will be sent to the corresponding author only. Please take a moment to check your funder requirements before choosing the appropriate license.

Additionally, JCB encourages authors to submit a short video summary of their work. These videos are intended to convey the main messages of the study to a non-specialist, scientific audience. Think of them as an extended version of your abstract, or a

short poster presentation. We encourage first authors to present the results to increase their visibility. The videos will be shared on social media to promote your work. For more detailed guidelines and tips on preparing your video, please visit https://rupress.org/jcb/pages/submission-guidelines#videoSummaries.

Thank you for your attention to these final processing requirements. Please revise and format the manuscript and upload materials within 14 days. If you need an extension for whatever reason, please let us know and we can work with you to determine a suitable revision period.

Please contact the journal office with any questions at cellbio@rockefeller.edu.

Thank you for this interesting contribution, we look forward to publishing your paper in Journal of Cell Biology.

Sincerely,

Ira Mellman, PhD
Monitoring Editor
Journal of Cell Biology

Dan Simon, PhD
Scientific Editor
Journal of Cell Biology

Reviewer #1 (Comments to the Authors (Required)):

The authors have addressed my concerns that the CAR are generating baseline signals, perhaps bebunking a myth based on early CARs that might have had more aggregation. They have also provided super-resolution videos of the process that have established the protrusions are dynamic individually, but are persistently generated at all time points and are not retraction fibres. Regarding the concerns about the value of the SLB platform, its the gold standard in the field for examination of F-actin dynamics (along with anti-CD3 coated substrates) and offers more opportunities to investigate cytoskeletal transport and F-actin dynamics as the ligands in the billayer can also be transported. So I feel like its a valuable addition to this line of research. Furthermore, I feel that it will open up further investigation of the mechanism, which was otherwise blocked by a lack of clear quantitative information. The fact that the CAR-T system they are using is in clinical trials also adds to the biological significance as a it builds on a highly refined synthetic biology effort.

Reviewer #3 (Comments to the Authors (Required)):

The authors of the submitted paper: "Super-resolution imaging reveals altered actin architecture at CAR-T cell immune synapses Actin reorganization at the CAR-T cell synapse" present data from a series of experiments aimed to compare actin organization in the immunological synapse (IS) formed as a result of T cell activation through synthetic chimeric antigen receptors (CARs) and through the T cell receptor (TCR). The authors first present an experiment used to determine the appropriate density of CAR ligands embedded in supported lipid bilayers (SLBs) necessary to activate pERK signaling in T cells. Once the ligand density was determined, the authors compared phenotype of actin in IS generated by T cells on SLBs following ligation of CARs vs. TCRs at three separate time points: 1, 3, and 8 minutes. In this way, the authors can assess dynamics of IS formation and general morphology at different time points. The authors describe a few phenotypic differences observed in their experiment, the main ones being an apparent delay in the maturation of actin phenotype in the CAR T cells, a lack of actin foci in the center of the IS of CAR T cells, differences in cortical density at the IS, and the presence of filopodial projections from the periphery of the IS in CAR T cells.
*With this iteration of the paper, the authors have added a live cell imaging experiment examining actin morphology spreading of CAR-transduced Jurkat cells over an antigen-embedded SLBs using a SoRa imaging system.
*I appreciate the addition of live-cell imaging experiment to this work. I wish the assay had been performed with some of the patient-derived transduced CD8+ T cells rather than CD4+ Jurkat cells. One note: the video file showing live imaging of the CAR-expressing cell appears to shift the Z plane about 3 minutes in. By changing the Z plane so dramatically, we aren't able to properly understand the progression of the actin phenotype at the synapse over time. I would like to see that this video is changed out with one that shows the actin morphology in a single plane over time. To my eye, it looks a little like the Jurkat cells expressing the CARs are simply not being activated well on these SLBs given the lack of spreading across the surface. I trust the authors have repeated this assay enough and are confident enough in the result to include it in the paper.
*My previous comments on the descriptive nature of the work remain, as no mechanistic or physiological study has been performed to give more details to the work. However, I accept the authors' response to this critique as adequate. To comment on the physiological effect of actin clearance on lytic granule release, the authors could perform killing assays, a degranulation assay (staining surface LAMP-1 after stimulation of cells on a planar surface or with targets) or live cell TIRF imaging of

lysosome secretion in T cells labeled with lysotracker or fluorescent protein-labeled LAMP-1. I do appreciate the removal of much of the conjecture of how observed actin phenotype could relate to CAR T cell functionality.
